# The Gut Microbiota as a Mediator of the Metabolic Benefits of Fungi and Their Polysaccharides: A Review of Evidence Addressing Causality

**DOI:** 10.3390/nu18193281

**Published:** 2026-10-06

**Authors:** Stevan Samardžić, Dragana D. Božić, Milica Drobac, Mirjana Marčetić, Jelena Arsenijević, Djordje Medarević, Zoran Maksimović

**Affiliations:** 1 Faculty of Pharmacy, Department of Pharmacognosy, University of Belgrade, Vojvode Stepe 450, 11221 Belgrade, Serbia; 2 Faculty of Pharmacy, Department of Microbiology and Immunology, University of Belgrade, Vojvode Stepe 450, 11221 Belgrade, Serbia; 3 Faculty of Pharmacy, Department of Pharmaceutical Technology and Cosmetology, University of Belgrade, Vojvode Stepe 450, 11221 Belgrade, Serbia; 4 Planet Systems Group d.o.o., Sestara Ninković 5, 21000 Novi Sad, Serbia

**Keywords:** obesity, diabetes, NAFLD, MASLD, dyslipidemia, fecal microbiota transplantation, FMT, antibiotic depletion, natural products, mushrooms

## Abstract

Prebiotic supplementation has emerged as a promising strategy to ameliorate metabolic disorders through beneficial reshaping of the gut microbiota. Accumulating evidence suggests that fungi and their polysaccharides (e.g., those from *Auricularia auricula-judae*, *Boletus edulis*, *Hirsutella sinensis*, *Isaria cicadae*, *Ramaria botrytoides*, and *Wolfiporia cocos*) possess considerable potential in this context. The present review summarizes studies providing data relevant to the potential involvement of the gut microbiota in mediating the metabolic benefits of fungal interventions. Observed outcomes, potentially linked to the gut microbiota, included reductions in body, visceral fat, and liver weights, improved lipid and glycemic profiles, enhanced insulin sensitivity, and decreased circulating lipopolysaccharide, leptin, and proinflammatory cytokines (TNF-α, IL-1β, IL-6). Upregulation of tight junction proteins in the colon implied strengthened gut barrier function. Histological analyses revealed smaller adipocytes and attenuation of hepatic steatosis. Additional effects included stimulation of white adipose tissue browning and enhanced hepatic thermogenesis. Alterations in gut microbiota composition were diverse; however, a reduction in the Firmicutes/Bacteroidetes ratio was commonly reported. Furthermore, oral administration of bacterial species enriched by fungal interventions, such as *Papillibacter cinnamivorans*, *Parabacteroides goldsteinii*, *Bacteroides intestinalis*, *Lactobacillus johnsonii*, and *Enterococcus casseliflavus*, reproduced the metabolic improvements, supporting their possible mediating role. Collectively, current evidence indicates that fungi and their polysaccharides may exert metabolic benefits, at least in part, through gut microbiota modulation; however, further investigation is needed to determine the extent to which the gut microbiota contributes causally to these benefits.

## 1. Introduction

Obesity and its related complications, including type 2 diabetes mellitus (T2DM) and nonalcoholic fatty liver disease (NAFLD), pose a major health burden worldwide. Recent estimates indicate that in 2022, 890 million adults were obese, along with 160 million children and adolescents aged 5–19 years. The number of people living with diabetes has sharply increased over the past three decades, reaching 830 million in 2022. Similarly, the global prevalence of NAFLD is alarmingly high, affecting roughly one-third of the population. If inadequately managed, these conditions can progress, substantially impairing quality of life and increasing the risk of premature death [1,2,3]. Consequently, substantial research efforts over recent decades have focused on elucidating the intricate molecular mechanisms underlying these diseases to improve therapeutic strategies. Recent findings highlight the gut microbiota as an important factor in metabolic health and disease, positioning it as a potential novel drug target. Various strategies have been proposed to beneficially modulate the gut microbiota or its metabolism, including phage therapy, supplementation with bio-engineered commensals, drugs targeting selected microbial metabolism, personalized nutrition, and administration of prebiotics, probiotics, synbiotics, or postbiotics [4,5,6]. Natural products hold significant but underexplored potential in this context. In particular, fungi and their polysaccharides have shown promising prebiotic properties. Although many studies report alterations in gut microbiota composition following these interventions, relatively few have investigated whether such changes are causally linked to the metabolic benefits observed in the host. Describing microbiota shifts alone is insufficient, as the microbial community is highly plastic and influenced by multiple factors, including diet. Experiments specifically designed to establish causality are therefore necessary.

This review aims to summarize current evidence on the positive metabolic effects of fungi and their polysaccharides that may be mediated by the gut microbiota, alongside the associated changes in microbial composition. Studies were selected based on experimental designs capable of assessing causality, including fecal microbiota transplantation (FMT), antibiotic depletion experiments, cohousing, and administration of specific bacterial strains previously shown to be enriched in treated animals.

## 2. Methods

The initial literature search was conducted in June 2025, and the final search was performed on 1 August 2026 using the Scopus, PubMed, and Web of Science databases. The search strategy was designed to identify studies examining the role of the gut microbiota in mediating the beneficial metabolic effects of fungal interventions and comprised four conceptual blocks: fungal interventions, gut microbiota, metabolic outcomes, and experimental designs suitable for exploring causal relationships. No publication date restrictions were applied. The complete search strategies for all three databases, the number of records retrieved from each database, the reasons for full-text exclusion, and the study selection flow diagram are provided in the Supplementary Materials (Table S1 and Figure S1).

Eligible studies were original research articles in English that involved mammalian subjects, investigated a fungal intervention, and employed an experimental design suitable for exploring a causal link between the gut microbiota and beneficial metabolic outcomes. Studies reporting only associations between changes in gut microbiota composition and metabolic outcomes, without experimental manipulation of the microbiota, were excluded.

For the purpose of this review, evidence was considered causality-oriented when a study used one or more gut microbiota manipulation approaches to examine whether the microbiota contributed to the beneficial metabolic effects of a fungal intervention. These approaches included antibiotic-mediated microbiota depletion, cohousing, fecal microbiota transplantation, and administration of specific bacteria previously shown to be enriched by the fungal intervention. Findings were considered supportive of causal involvement when beneficial metabolic effects were lost or attenuated following antibiotic treatment or when the beneficial metabolic phenotype was transferred or reproduced through FMT, cohousing, or administration of specific bacteria. Conversely, findings were considered non-supportive when the effects persisted after antibiotic treatment or when these approaches failed to transfer or reproduce the beneficial phenotype. In this review, microbiota dependence refers to attenuation or loss of the beneficial metabolic effects following antibiotic-mediated microbiota depletion. Differences in the strength of the evidence relevant to assessing causality were considered in the Discussion Section. Inclusion in this review does not imply that causality was definitively established, but rather that the study provided evidence relevant to its assessment.

Records were merged and deduplicated in Microsoft Excel using DOIs and, when these were unavailable, article titles. Titles and abstracts were screened first, followed by full-text assessment. The literature search, screening, data extraction, and recording of reasons for full-text exclusion were performed by S.S.

## 3. Results

In total, the search yielded 1545 records. After removing 393 duplicates, 1152 unique records remained. Following initial screening and detailed eligibility assessment, 28 causality-oriented articles met all eligibility criteria. One complementary article, although not causality-oriented, was also retained, as it provided data necessary to interpret the results of one of these studies. The remaining 1123 records were excluded. Ultimately, 29 articles were included in this review. The study selection flow diagram is given in the Supplementary Materials (Figure S1).

Of the twenty-eight eligible articles, four focused on *Aspergillus cristatus*, four on *Ganoderma lucidum*, three on *Wolfiporia cocos*, two on *Auricularia auricula-judae*, and two on *Cordyceps militaris*, while each of the remaining articles examined a different fungal species. Mice were the predominant experimental species, and high-fat diet-induced obesity was the most frequently employed experimental model. All included articles were published between 2015 and 2026, with most appearing in the last several years.

FMT was the predominant experimental approach and was employed in 21 of the 28 eligible articles. In these studies, fungal interventions were most frequently administered to donor animals at doses ranging from 100 to 400 mg/kg/day. Eight weeks was the most common duration of the experimental period in recipient animals, although shorter and longer periods were reported. In antibiotic-depletion experiments, fungal interventions were generally administered at doses ranging from 200 to 400 mg/kg/day. Experiment duration ranged from 6 to 12 weeks, with 8 weeks being the most common. Specific bacteria were administered at doses ranging from 4 × 10^7^ to 1 × 10^9^ CFU, and these experiments lasted either 6 or 8 weeks. All fungal and bacterial interventions were administered orally.

The summarized findings of the literature review are presented in Table 1, which provides data from studies investigating the following fungal species: *Aspergillus cristatus* (#1–#4), *Auricularia auricula-judae* (#5, #6), *Boletus edulis* (#7), *Cordyceps militaris* (#8, #9), *Ganoderma lucidum* (#10–#13), *Inonotus obliquus* (#14), *Isaria cicadae* (#15), *Lactifluus volemus* (#16), *Lyophyllum decastes* (#17), *Morchella esculenta* (#18), *Ophiocordyceps sinensis* (#19), *Phellinus igniarius* (#20), *Ramaria botrytoides* (#21), *Schizophyllum commune* (#22), *Sparassis latifolia* (#23), *Trametes versicolor* (#24), *Tremella fuciformis* (#25), and *Wolfiporia cocos* (#26–#28). In the subsequent text, the relevant information is organized according to the examined fungal species.

*Aspergillus cristatus* (syn.: *Eurotium cristatum*) is a dominant microorganism in Fuzhuan tea (also known as Fu brick tea), contributing to its characteristic taste [8]. Several studies have investigated the potential metabolic benefits of this fungus in the context of obesity and T2DM. Lu et al. (2025) examined the effects of purified water-soluble galactomannan isolated from mature spores of *A. cristatus* in high-fat diet (HFD)-fed mice and confirmed its biological activity, prompting a follow-up FMT experiment. Mice receiving fecal microbiota from galactomannan-treated obese donors exhibited marked metabolic improvements compared with those receiving microbiota from saline-treated obese donors. Specifically, they showed reduced body weight, liver steatosis and liver weight, inguinal and epididymal fat weights, and adipocyte size. In addition, glycemic control, lipid profile, and insulin sensitivity were improved. In the galactomannan-treated animals, the gut microbiota was characterized by a lower Firmicutes/Bacteroidetes ratio, increased *Parabacteroides*, and decreased *Lachnoclostridium* [7]. Consistent results were obtained when obese mice were supplemented with live *A. cristatus* isolated from Fuzhuan tea. The microbiota of *A. cristatus*-treated HFD-fed mice was sufficient to confer similar benefits: when transferred to HFD-fed recipients, it reduced body, inguinal fat, epididymal fat, and liver weights, and improved glucose homeostasis and serum lipid profile. Treatment with live *A. cristatus* significantly modulated gut microbiota composition, leading to reduced *Lactobacillus reuteri* and increased *Bacteroides acidifaciens*, *Blautia coccoides*, and *Faecalibacterium prausnitzii* [8]. In another study, purified polysaccharides from sporoderm-broken cleistothecia of *A. cristatus* were tested in obese rats. Again, the beneficial effects were transmissible via microbiota transfer, suggesting a causal role of gut microbes. Recipients of microbiota from treated donors exhibited reduced body, epididymal fat, and perirenal fat weights, smaller epididymal adipocytes, lower liver/body weight ratio, decreased hepatic steatosis, and reduced serum liver enzyme concentrations (AST and ALT). Insulin resistance and serum lipid levels were improved. In the colon, goblet cell number and expression of tight junction proteins were increased, while serum LPS and proinflammatory cytokines were decreased, and the IL-10 level was elevated. In both recipient and donor rats, the gut microbiota was markedly modulated, with altered levels of *Akkermansia*, *Bacteroides*, *Romboutsia*, *Blautia*, and *Desulfovibrio* [9]. Finally, crude extracellular polysaccharides produced by *A. cristatus* isolated from Fu brick tea were evaluated in a mouse model of T2DM. Under antibiotic treatment, their beneficial effects on glucose tolerance, insulin sensitivity, serum lipid profile, serum glucagon-like peptide-1 level, hepatic mRNA expression of *Ahr* and *Tsc2*, and hepatic mTORC1 content were no longer observed, supporting the involvement of gut microbiota in these effects. Polysaccharide administration also decreased the relative abundance of norank_f__Eubacterium_coprostanoligenes_group in HFD-fed diabetic mice compared with saline-treated HFD-fed diabetic controls [10].

*Auricularia auricula-judae* (syn.: *Auricularia auricula*) is a widely distributed edible mushroom, particularly common across northern temperate regions. It has been consumed worldwide and has a more than 1000-year-long history of use in oriental medicine. Current evidence indicates that polysaccharides represent its primary bioactive constituents [11,12]. To investigate the potential of *A. auricula-judae* polysaccharides to alleviate obesity and related metabolic disorders, Zong et al. (2023) conducted experiments in HFD-fed mice. The obtained results were promising, triggering further assessment of gut microbiota involvement using complementary approaches. First, the anti-obesity effect was abolished in mice treated concurrently with antibiotics and *A. auricula-judae* polysaccharides, indicating that the gut microbiota plays a crucial mediating role. Second, FMT from polysaccharide-treated donors to HFD-fed recipients conferred significant metabolic benefits. Recipient mice displayed reduced body weight, smaller abdominal fat pad, improved blood lipid profile, lower hepatic triglyceride levels, and better glycemic control. Histological analysis revealed reduced adipocyte size, while molecular analysis showed decreased expression of adipogenesis-related genes. Examination of the fecal microbiota in mice treated with the polysaccharides showed decreased relative abundance of *Mucispirillum*, alongside increased relative abundances of *Peptococcus*, *Muribaculum*, *Anaerovorax*, and *Papillibacter*. Correlation analysis suggested that *Papillibacter cinnamivorans* might be a key contributor to these effects. To verify this, mice were administered a commercial *P. cinnamivorans* strain, which reproduced the major outcomes observed with whole fecal transplants. Body, liver, and abdominal fat weights were reduced; adipocyte size and expression of lipid metabolism-related genes were decreased; and hepatic and serum triglyceride levels and serum cholesterol levels were lowered. Blood glucose level also declined relative to the control group. Additional assessment revealed diminished signs of colonic inflammation and reduced colonic proinflammatory cytokines. Intestinal barrier function was improved, accompanied by lower serum LPS levels. At the cellular level, activation of JAK–STAT signaling was suppressed. Furthermore, intestinal lipid absorption was reduced, and hepatic thermogenesis was enhanced. Taken together, these results suggest that *P. cinnamivorans* may serve as a promising next-generation probiotic for obesity management, while *A. auricula-judae* polysaccharides may act as prebiotics [11]. In another study, Zhou et al. (2023) evaluated *A. auricula-judae* polysaccharides in HFD-induced obese mice and obtained similar results. Following FMT, the involvement of gut microbiota was supported, as metabolic disturbances were improved in recipient animals. Reductions were observed in body and liver weights, as well as in the relative weights of epididymal, mesenteric, and perirenal adipose tissues. Blood glucose, blood lipids, and hepatic enzyme levels were decreased, and insulin sensitivity was enhanced. The intestinal barrier function was strengthened, while serum LPS, TNF-α, and IL-6 concentrations were all reduced. At the signaling level, modulation of the TLR4/JNK pathway was observed. Additionally, fecal samples from recipient mice exhibited higher concentrations of short-chain fatty acids (SCFAs). The gut microbiota composition also shifted, as treatment with *A. auricula-judae* polysaccharides increased relative abundances of SCFA-producing genera such as *Allobaculum*, *Roseburia*, and *Anaerotruncus* [12].

*Boletus edulis*, also known as king bolete, is an edible mushroom that contains flavonoids, vitamins, proteins, and polysaccharides. In a recent study by Zhao et al. (2025), the metabolic effects of a purified low-molecular-weight polysaccharide from *B. edulis* were examined in HFD-fed mice. The FMT experiment indicated that the gut microbiota plays a role in mediating its beneficial effects, which included reduced body weight, improved serum glucose and lipid profiles, decreased adipose tissue mass and hepatic lipid accumulation, and lower serum levels of liver enzymes (AST and ALT). Additionally, treatment decreased serum concentrations of TNF-α and LPS, while increasing colonic levels of occludin and mucin-2. Further analysis revealed an improvement in TLR4/MAPK-mediated disturbances in the hepatic expression of proteins involved in glucose and lipid metabolism. Administration of the purified *B. edulis* polysaccharide to HFD-fed mice also modified gut microbiota composition: beneficial bacteria such as members of the families S24-7 and Lachnospiraceae, as well as the genera [*Prevotella*] and *Lactobacillus*, were enriched, whereas potentially harmful taxa, particularly *Desulfovibrio*, were reduced. Alterations were also observed in recipient mice following FMT. Specifically, when feces from HFD-fed mice treated with the *B. edulis* polysaccharide were transplanted into HFD-fed recipients, the relative abundance of *Firmicutes* decreased and that of *Bacteroidetes* increased, compared with recipients gavaged with feces from saline-treated HFD-fed donors. At the genus level, *Lactobacillus* and *Bacteroides* were enriched, while *Allobaculum* and *Desulfovibrio* decreased in abundance [13].

*Cordyceps militaris* has long served as both a traditional medicine and a functional food in East Asia. Zhao et al. (2023) evaluated this fungus in a model of HFD- and streptozotocin-induced diabetes in mice. The positive effects of *C. militaris* neutral refined polysaccharide were transferable via FMT. The experimental design comprised test and control recipient groups; the former received fecal material from *C. militaris* polysaccharide-treated diabetic mice, whereas the latter received PBS. Recipient test animals showed reduced water and food intake, improved glycemic and lipid profiles, and increased insulin sensitivity. Serum liver enzyme levels and colonic proinflammatory cytokine concentrations were decreased, while colonic tight junction proteins were increased, indicating enhanced intestinal barrier function. Endotoxemia was attenuated. Histopathological examination revealed reduced colon and pancreas damage compared with controls. At the signaling level, the colonic TLR4/NF-κB pathway was inhibited. The gut microbiota composition of *C. militaris* polysaccharide-treated diabetic mice was also reshaped, with increased relative abundances of *Alistipes*, Lachnospiraceae_NK4A136_group, and norank_f_Muribaculaceae, and decreased relative abundance of *Enterococcus* [14]. In a study by Cai et al. (2026), *C. militaris* polysaccharide (CMP) ameliorated HFD-induced obesity and associated metabolic disturbances in mice. Its beneficial effects were preserved in animals receiving an antibiotic cocktail that did not contain neomycin but were lost for most outcomes following treatment with either neomycin or a broad-spectrum cocktail containing neomycin. The affected outcomes included body weight gain, serum lipid parameters (total cholesterol, triglycerides, and low-density lipoprotein cholesterol), blood glucose and serum insulin levels, hepatic steatosis and inflammation, intestinal barrier injury, and serum LPS. Further analysis suggested that brassicasterol may contribute to these effects, potentially through the upregulation of hepatic apolipoprotein A-IV gene expression. CMP administration decreased the relative abundance of Firmicutes and increased that of Bacteroidetes. At the genus level, *Parabacteroides* and *Lactobacillus* were enriched, while the relative abundance of *Parabacteroides goldsteinii* increased at the species level [15].

*Ganoderma lucidum* is a medicinal mushroom with a long history of traditional use and is believed to promote health and longevity. Its biological effects have been attributed to triterpenes, polysaccharides, and proteoglycans. Chang et al. (2015) investigated the effects of a water extract of *G. lucidum* mycelium (WEGL) on obesity in HFD-fed mice, as well as the role of gut microbiota in mediating these effects. FMT from WEGL-treated donors conferred metabolic benefits to recipient mice, including reductions in body weight, epididymal and subcutaneous fat mass, and liver weight. Additionally, mRNA expression of proinflammatory cytokines in liver and adipose tissue was decreased, while tight junction protein mRNA expression in the ileum was upregulated, suggesting enhanced intestinal barrier integrity. Lipogenic gene expression in liver and adipose tissue was suppressed. WEGL supplementation reversed HFD-induced dysbiosis, as indicated by a decreased Firmicutes/Bacteroidetes ratio and a reduced relative abundance of endotoxin-bearing Proteobacteria [16]. Beyond the mycelium, *G. lucidum* polysaccharides derived from sporoderm-broken spores were studied by Sang et al. (2021). An FMT experiment demonstrated that fecal microbiota from polysaccharide-treated mice transmitted positive metabolic effects, including reductions in body weight and epididymal and inguinal white adipose tissue mass. Serum levels of LPS and the proinflammatory cytokine TNF-α were also lowered. In HFD-fed mice, administration of polysaccharides from *G. lucidum* spores resulted in a nonsignificant reduction in the Firmicutes/Bacteroidetes ratio [17]. Extending their previous findings, Sang et al. (2026) examined polysaccharides from sporoderm-broken *G. lucidum* spores. Antibiotic intervention abolished their beneficial effects on body weight gain, white adipose tissue mass, glucose tolerance, adipocyte hypertrophy and macrophage infiltration in adipose tissue, ileal goblet cell number, ileal FABP4 and PPARγ protein expression, serum triglycerides, and lipopolysaccharide-binding protein. Microbiota profiling of obese mice receiving the polysaccharides indicated increased relative abundances of *Lactobacillus* and *Bifidobacterium*, with significant enrichment of *L. johnsonii* and *L. reuteri* at the species level. Oral administration of *L. johnsonii* produced a range of beneficial metabolic effects consistent with those observed following the administration of *G. lucidum* polysaccharides. Specifically, *L. johnsonii* administration reduced body weight gain and white adipose tissue mass, attenuated adipocyte hypertrophy and macrophage infiltration in white adipose tissue, increased adipose triglyceride lipase protein expression, improved glycemic control, and strengthened intestinal barrier integrity. The accompanying increase in fecal butyrate suggested that this metabolite may contribute to the effects of *L. johnsonii* [18]. In a recent study by Zhao et al. (2026), a purified fraction of enzyme-hydrolyzed polysaccharides from *G. lucidum* fruiting bodies was tested in an HFD-induced mouse model of NAFLD. The beneficial effects were transmissible via FMT. Specifically, recipient mice showed reduced serum levels of AST, ALT, and LPS, as well as decreased hepatic lipid accumulation. At the molecular level, suppression of the TLR4/NF-κB/MAPK pathway indicated anti-inflammatory activity. Gut microbiota analysis revealed that, at the genus level, administration of the fraction increased the levels of *Lachnoclostridium* and *Lactobacillus*, while decreasing those of *Akkermansia*, Escherichia-Shigella, *Blautia*, *Romboutsia*, and *Odoribacter* [19].

*Inonotus obliquus* is an edible parasitic fungus that is found mainly on birch trees in cold regions and has a history of medicinal use. Its constituents include polysaccharides, triterpenoids, and polyphenols. Sun et al. (2026) investigated its effects in HFD-induced obese mice using a combined extract obtained through three hot reflux extractions with 90% and 100% ethanol and subsequently conducted an FMT experiment. HFD-fed recipients of fecal microbiota from extract-treated HFD-fed donors exhibited lower body and liver weights and reduced masses of brown, subcutaneous white, and epididymal white adipose tissues compared with recipients of microbiota from saline-treated HFD-fed donors. Histological analysis revealed reduced lipid accumulation in adipose tissues, attenuated adipocyte hypertrophy, and reduced hepatic steatosis. During cold exposure, recipient mice maintained a higher rectal temperature. Protein levels of UCP1 and key oxidative phosphorylation subunits were increased in brown and subcutaneous white adipose tissues, together with increased mRNA expression of thermogenic genes in both tissues. *Enterococcus casseliflavus* was identified as a potential mediator of the effects of the extract, leading to further experiments with this species. Oral *E. casseliflavus* administration reduced body and liver weights, adipose tissue masses and lipid accumulation, while improving glucose tolerance, insulin sensitivity, and the serum lipid profile. Colonic goblet cell number and intestinal tight junction protein levels were increased, whereas inflammatory cell infiltration and serum LPS were reduced. The bacterium also increased mouse core body temperature after acute cold exposure, thermogenic gene mRNA expression, and protein levels of UCP1 and key oxidative phosphorylation subunits in brown and subcutaneous white adipose tissues. Following a single oral dose, *E. casseliflavus* did not stably colonize the gut. Nevertheless, repeated administration decreased the Firmicutes/Bacteroidetes ratio in HFD-fed mice [20].

*Isaria cicadae* (syn.: *Cordyceps cicadae*), a fungus parasitizing cicada larvae, has been used as a tonic food and traditional medicine for heart palpitations, infantile convulsions, chronic kidney diseases, and eye disorders. Numerous beneficial effects have been attributed to this fungus, including antioxidant, anti-inflammatory, and blood glucose-regulating activities. Polysaccharides are considered one of its major bioactive constituents. The role of gut microbiota in mediating the metabolic effects of *I. cicadae* crude polysaccharides was investigated using an FMT approach. Diabetic recipient mice were allocated into either a test group, which received fecal material from *I. cicadae*-treated diabetic donors, or a control group, which received saline. Test group recipients exhibited improved blood glucose control, enhanced insulin sensitivity, and reduced water intake and urinary output. Treatment with the polysaccharides substantially altered gut microbiota composition, as reflected by the decreased Firmicutes/Bacteroidetes ratio, increased relative abundances of *Bacteroides*, *Odoribacter*, *Alloprevotella*, *Parabacteroides*, and *Mucispirillum*, and reduced relative abundances of *Helicobacter* and *Lactobacillus* [21].

*Lactifluus volemus* (syn.: *Lactarius volemus*) is an edible mushroom valued for its nutritional qualities. Its polysaccharides have previously been reported to exhibit noteworthy biological activities, including antiproliferative and immunomodulatory effects. In continuing efforts to pharmacologically characterize these compounds, Xu et al. (2025) examined crude polysaccharides obtained from *L. volemus* fruiting bodies for their ability to attenuate high-fat/high-fructose diet-induced obesity and related metabolic disturbances in mice. Beneficial effects were observed, with simultaneous antibiotic treatment abolishing improvements in several parameters, including adipose tissue weight, serum lipid profile, glycemia, and insulin sensitivity. The gut microbiota of mice gavaged with crude polysaccharides, compared with that of control mice, was characterized by a lower Firmicutes/Bacteroidetes ratio, increased relative abundances of *Bacteroides*, *Akkermansia*, *Bifidobacterium*, and *Lactobacillus*, and decreased abundance of *Parasutterella* [22].

*Lyophyllum decastes* is a mushroom traditionally used in East Asia both as a food and for its medicinal properties. Its antioxidant, antidiabetic, and hypolipidemic effects have been documented in multiple studies. Oral administration of *L. decastes* polysaccharides exerted beneficial effects in HFD-induced obese mice. The composition of cecal microbiota was altered by the intervention, as evidenced by increased relative abundances of *Bacteroides intestinalis* and *Lactobacillus johnsonii*. To determine whether these effects were microbiota-dependent, mice were orally gavaged with *B. intestinalis*, *L. johnsonii*, or a combination of both strains. The experiments implied that these bacteria contributed to the observed metabolic benefits. Specifically, body weight and the weights of total, subcutaneous, and mesenteric white adipose tissue were reduced. Plasma and liver lipid levels were lowered, and serum liver enzyme levels decreased. Additionally, administration of these bacterial species stimulated brown adipose tissue thermogenesis and promoted browning of subcutaneous white adipose tissue [23].

*Morchella esculenta* is a commercially important mushroom, valued for its unique flavor and nutritional properties. Its polysaccharides (MEP) have shown several biological activities in preclinical studies, including antioxidant, anti-inflammatory, and immunomodulatory effects [24]. Given the previously demonstrated beneficial metabolic outcomes of MEP in HFD-induced obese mice [24], Zang et al. (2025) examined whether these improvements depend on an intact gut microbiota [25]. No significant differences in weight gain rate, glucose tolerance, epididymal fat and liver indices, or serum liver enzyme levels were observed between HFD + saline + antibiotics and HFD + MEP + antibiotics groups [25], suggesting the role of the intestinal microbial community. In the study by Liu et al. (2023), MEP showed the ability to modify gut microbiota, as indicated by the decreased Firmicutes/Bacteroidetes ratio, increased abundance of *Lactobacillus* and *Dubosiella*, and decreased abundance of *Faecalibaculum* [24].

The medicinal fungus *Ophiocordyceps sinensis* (caterpillar fungus) and its anamorph *Hirsutella sinensis* have been used in traditional medicine for immunomodulatory properties. The water extract of *H. sinensis* mycelium (HSM) and its high-molecular-weight polysaccharide fraction (H1, >300 kDa) were examined in a murine model of HFD-induced obesity. The involvement of gut microbiota in mediating the pharmacological effects was investigated using multiple approaches. Treatment of mice with antibiotics revealed that neomycin abolished the anti-obesogenic effects of H1. Furthermore, ex vivo antibiotic treatment of fecal samples, combined with an FMT experiment, demonstrated that ex vivo neomycin exposure also eliminated the anti-obesogenic activity of H1. Taken together with the microbiota composition analysis, these results indicated that neomycin-sensitive bacteria, including *Parabacteroides goldsteinii*, might be responsible for the observed activity. An FMT experiment further supported the causal role of gut microbiota in the anti-obesogenic effects. Transplantation of feces from HFD-fed mice treated with HSM or H1 into recipient HFD-fed mice produced several metabolic improvements, including reduced body weight gain, visceral fat pad mass, and adipocyte size, alongside enhanced insulin sensitivity. Serum levels of proinflammatory cytokines and endotoxin were decreased, intestinal permeability was reduced, and liver damage was alleviated, as indicated by a lower NASH activity index score. Oral administration of live *P. goldsteinii* to mice produced effects consistent with those observed following HSM and H1 treatments. Moreover, *P. goldsteinii* was shown to induce thermogenesis and regulate hepatic gene expression related to lipid transport, lipogenesis, and β-oxidation. Heat-killed or pasteurized *P. goldsteinii* failed to reduce body weight or visceral fat accumulation. Collectively, these findings suggest that high-molecular-weight polysaccharides from HSM may act as prebiotic candidates for the treatment of obesity and type 2 diabetes, while *P. goldsteinii* may represent a probiotic candidate for managing obesity and associated metabolic disorders [26].

*Phellinus igniarius* (“forest gold”) is an edible and medicinal mushroom traditionally used to treat gastrointestinal disorders and promote blood circulation. Its polysaccharides were investigated by Ni et al. (2023) in mice with diabetes induced by an HFD and streptozotocin. The FMT approach provided evidence for the important role of gut microbiota in mediating the biological effects of these polysaccharides. Diabetic mice receiving fecal transplants from donor diabetic mice treated with *P. igniarius* polysaccharides showed improvements compared to diabetic controls receiving saline. Specifically, water intake and urinary output were reduced, while blood glucose and insulin levels were lower. Circulating proinflammatory cytokine concentrations also decreased, and histopathological changes in the pancreas, liver, and kidney were alleviated. Moreover, gut dysbiosis was ameliorated, accompanied by a significant increase in the relative abundance of *Lactobacillus* [27].

*Ramaria botrytoides* is a mushroom recognized as a rich source of compounds with antibacterial, antioxidant, anti-inflammatory, and immunoregulatory properties. It has traditionally been used as both a tonic food and a medicine. Its purified polysaccharide was evaluated in diabetic mice, and the role of gut microbiota in mediating its effects was investigated through an FMT experiment. Compared with saline-treated diabetic control mice, recipients of fecal material from the intervention group exhibited an improved metabolic phenotype. Food and water intake, as well as urinary output, were reduced, while blood glucose and insulin levels were lowered. Histopathological alterations in the pancreatic islets and liver were alleviated, and hepatic lipid accumulation was inhibited. Treatment with *R. botrytoides* polysaccharide enriched several bacterial genera, including *Alistipes*, *Bacteroides*, *Ruminococcus*, *Odoribacter*, *Akkermansia*, and *Turicibacter* [28].

*Schizophyllum commune* fruiting bodies are edible and known to contain polysaccharides, polyphenols, terpenoids, and organic acids. In a recent study, Yin et al. (2026) demonstrated beneficial effects of its polysaccharide in HFD-fed animals and showed that the anti-obesogenic activity was transferable via FMT. In HFD-fed recipient mice gavaged with fecal material from *S. commune* polysaccharide-treated HFD-fed donors, a wide range of metabolic improvements was observed compared with the control group. Specifically, body weight and the relative weights of the liver and epididymal white adipose tissue were reduced; serum and hepatic lipid profiles were improved; and levels of proinflammatory cytokines in liver and serum decreased. Expression of ZO-1 mRNA was increased, whereas serum LPS concentration was reduced. Furthermore, the hepatic malondialdehyde level and serum AST and ALT levels were lower, and the number and size of lipid droplets in the liver and epididymal adipocytes decreased. The gut microbiota composition shifted in response to the intervention and after FMT treatment. The notable change in both recipient and donor mice was an increased relative abundance of *Faecalibaculum rodentium*, suggesting that this bacterial species may contribute to the beneficial metabolic effects [29].

The edible and medicinal fungus *Sparassis latifolia* contains polysaccharides with reported antioxidant, immunomodulatory, and anti-inflammatory activities. These polysaccharides improved glucose regulation in mice with hyperglycemia induced by a high-fat/high-sugar diet and streptozotocin. The potential involvement of the gut microbiota was examined using cohousing. Saline-treated diabetic mice cohoused with polysaccharide-treated diabetic mice exhibited lower fasting blood glucose, lower blood glucose at 2 h, a smaller area under the curve in the oral glucose tolerance test, and reduced intestinal crypt depth, compared with separately housed diabetic controls, supporting the transmissibility of these favorable outcomes. Fecal butyric acid and total short-chain fatty acid concentrations were higher in the cohoused saline-treated diabetic mice than in separately housed diabetic controls. Polysaccharide administration significantly reversed the elevated Firmicutes/Bacteroidetes ratio observed in diabetic controls [30].

*Trametes versicolor* (syn.: *Coriolus versicolor*) is traditionally used in Asia for the prevention and treatment of cancer and infectious diseases. Li et al. (2019) investigated the effects of its purified protein-bound β-glucan, isolated from the fruiting body, in mice with HFD-induced obesity. An FMT experiment demonstrated that the beneficial effects were transferable, as recipient mice inoculated with fecal material from protein-bound β-glucan-treated donors exhibited reduced body weight, lower serum leptin and proinflammatory cytokine levels, and increased serum adiponectin. In terms of gut microbiota composition, treatment with the protein-bound β-glucan increased the relative abundance of *Akkermansia muciniphila*, although it did not reverse the HFD-induced shift in the Firmicutes/Bacteroidetes ratio [31].

*Tremella fuciformis* has been used for thousands of years in traditional Chinese medicine and is also valued for its unique flavor. Modern research has highlighted the beneficial effects of its polysaccharide, including immunomodulatory, anti-aging, and antioxidant properties, as well as attenuation of atopic dermatitis. The polysaccharide from *T. fuciformis* was tested in HFD-induced obese mice to evaluate its efficacy and mechanisms of action. FMT suggested that the observed metabolic improvements were microbiota-dependent. Specifically, epididymal and perirenal fat masses, as well as liver weight, were reduced; lipid and glycemic profiles were improved, and serum levels of glucagon-like peptide-1 and -2 were increased. The intervention also modulated gut microbiota. In the donor mice, the Firmicutes/Bacteroidetes ratio was reduced, and at the genus level, the relative abundance of *Alistipes* increased, whereas those of *Desulfovibrio* and *Bilophila* decreased. Similarly, in recipient mice, the Firmicutes/Bacteroidetes ratio decreased [32].

*Wolfiporia cocos* (syn.: *Poria cocos*) is a fungus that parasitizes the roots of pine trees [34]. It is used in traditional Chinese medicine for the treatment of chronic gastritis, gastric atony, edema, and dizziness, and is also consumed as a food believed to exert beneficial metabolic effects. In a study by Sun et al. (2019), the focus was a water-insoluble polysaccharide obtained from the sclerotium of *W. cocos*. Its beneficial effects were demonstrated in ob/ob mice, and the involvement of gut microbiota was investigated using FMT. The test recipient mice showed improved insulin sensitivity, reduced lipids and glucose levels, and decreased weight gain compared with the control recipient mice, which received vehicle. Analysis of the cecal microbiota revealed enrichment of butyrate-producing bacteria from the family Lachnospiraceae and genus *Clostridium* [33]. In a subsequent study, Liu et al. (2025) examined the water-soluble polysaccharides of *W. cocos* (WCP). WCP treatment produced positive effects in HFD-induced obese mice, which were also reproduced in HFD-fed animals after FMT from WCP-treated donors. Administration of fecal matter from WCP-treated animals led to reductions in body weight, serum lipid and leptin levels, as well as a lower leptin-to-adiponectin ratio. Insulin sensitivity and epididymal adipocyte morphology were improved. The expression of proinflammatory cytokine mRNAs in epididymal adipose tissue and colon was attenuated, while expression of tight junction protein claudin-1 in the colon increased, indicating an enhancement of intestinal barrier integrity. Correspondingly, serum LPS levels were lower than those in the control group. Fecal analysis revealed elevated SCFA concentrations. The authors attributed the positive effects to modulation of the FGF21-PI3K/AKT signaling pathway. Microbiota profiling showed increased relative abundances of *Lactobacillus*, *Allobaculum*, and *Phascolarctobacterium* [34]. Because *W. cocos* polysaccharides have relatively low water solubility, Zhu et al. (2022) prepared related oligosaccharides (WCO) by enzymatic hydrolysis, obtaining a product with improved solubility and a lower degree of polymerization (2–6). WCO failed to restore glucolipid metabolism in antibiotic-treated mice, indicating a microbiota-dependent mechanism. In an FMT experiment, the transfer of fecal microbiota from WCO-treated donors resulted in reduced body and pancreas weights in recipient mice. At the phylum level, the gut microbiota of HFD-fed mice treated with WCO showed decreased relative abundance of Firmicutes and increased relative abundance of Bacteroidetes. Several genera were enriched, including *Lactobacillus*, whereas others such as *Desulfovibrio* were suppressed. Recipient mice receiving feces from WCO-treated donors exhibited higher Shannon diversity, increased relative abundances of *Bifidobacterium*, *Desulfovibrio*, and *Bacteroides*, and decreased relative abundances of *Staphylococcus*, *Roseburia*, and Lachnospiraceae_NK4A136_group [35].

**Figure 1:**
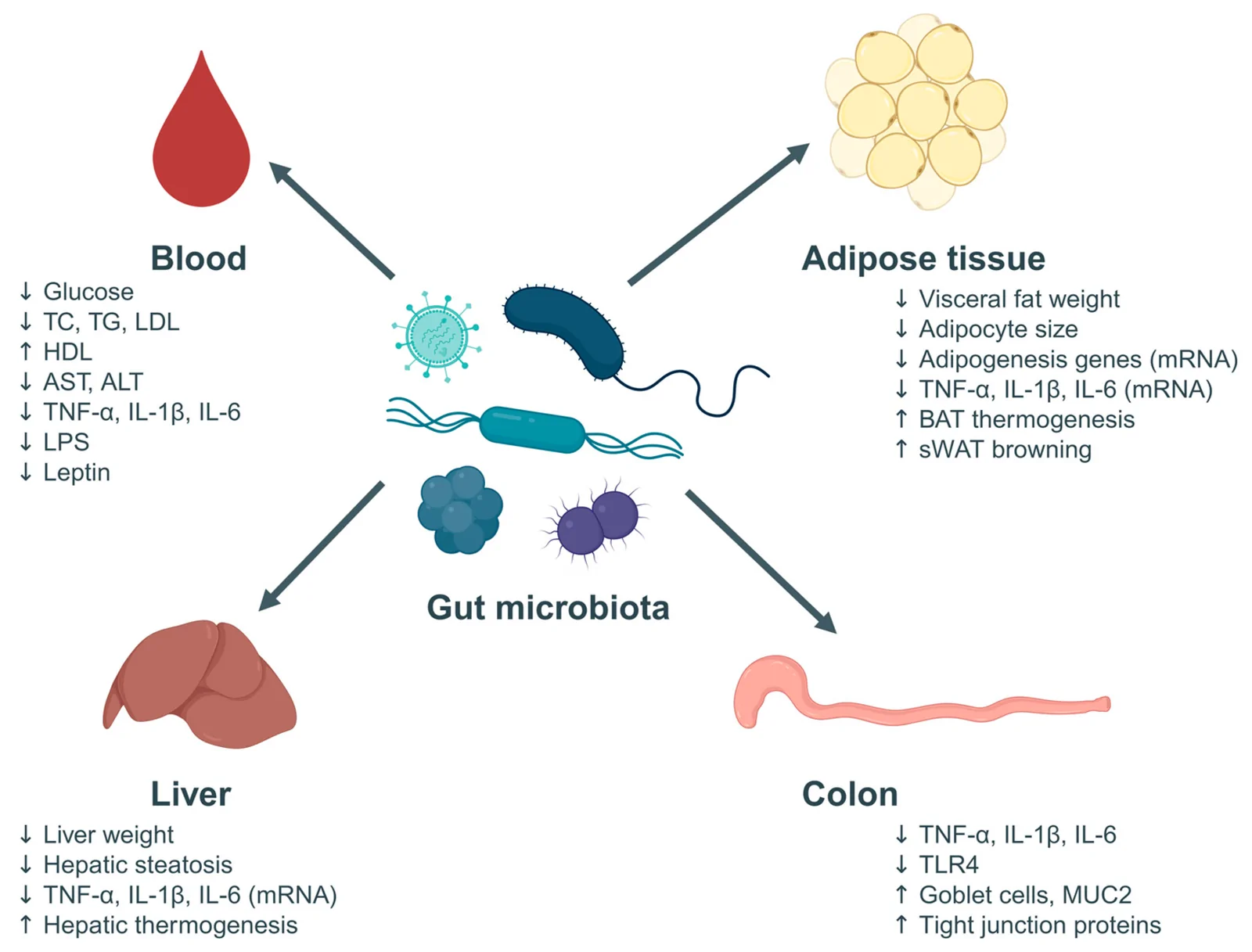
The metabolic and related effects of fungi and their polysaccharides that may depend on the gut microbiota. Abbreviations: ALT, alanine aminotransferase; AST, aspartate aminotransferase; BAT, brown adipose tissue; HDL, high-density lipoprotein; IL-1β, interleukin-1 beta; IL-6, interleukin-6; LDL, low-density lipoprotein; LPS, lipopolysaccharide; MUC2, mucin 2; sWAT, subcutaneous white adipose tissue; TC, total cholesterol; TG, triglyceride; TLR4, toll-like receptor 4; TNF-α, tumor necrosis factor-alpha. Created in BioRender. center, T. T. (2026) https://BioRender.com/evlfg4n.

The summarized findings of the literature review are illustrated in Figure 1.

## 4. Discussion

### 4.1. Potential Mechanisms Linking Gut Microbiota Modulation to Metabolic Benefits

Across the causality-oriented studies included in this review, several changes in host tissues provide potential mechanistic links between modulation of the gut microbiota and the beneficial metabolic effects of fungal interventions. Mechanistic investigations have focused primarily on the intestine, liver, and adipose tissue. The evidence summarized in this section was obtained from studies employing gut microbiota manipulation approaches, including FMT, antibiotic depletion, cohousing, and administration of specific bacteria.

Enhancement of the intestinal barrier emerged as one of the most consistently reported effects. Increased numbers of goblet cells were observed in the ileum [18] and colon [9,20], together with increased colonic mucin-2 content [13]. Multiple studies also reported increased expression of colonic tight-junction proteins, including occludin, zonula ocludens-1, and claudin-1 [9,11,14,34], as well as decreased colonic levels of proinflammatory cytokines IL-6, TNF-α, and IL-1β [11,14]. At the signaling level, suppression of HFD-induced intestinal inflammation was accompanied by modulation of the JAK-STAT pathway [11], while inhibition of colonic TLR4/NF-κB signaling was reported in another study [14]. Additional intestinal effects included modulation of ileal FABP4-PPARγ signaling [18] and reduced mRNA expression of key lipid transporters in colon and jejunum, including *Mtp* and *Fatp4*, which may contribute to lower serum triglycerides, cholesterol, and free fatty acid levels [11].

Strengthening of the mucus and epithelial barrier, together with the attenuation of intestinal inflammation, could limit the translocation of gut-derived LPS into the systemic circulation. This may be metabolically relevant because endotoxemia can induce chronic low-grade inflammation and thus contribute to insulin resistance and dysfunction of the liver and adipose tissue. Consistent with this proposed mechanism, reduced serum LPS levels were documented in numerous studies [9,11,12,13,14,17,20,26,29,31,34]. Reductions in circulating monocyte chemoattractant protein-1 [9], TNF-α, IL-6, and IL-1β [9,11,12,13,17,26], as well as leptin [31,34], were also reported.

The liver represents another major site of potentially microbiota-mediated effects. Reductions in hepatic lipid accumulation were observed across multiple studies [7,9,11,13,19,20,23,28,29], while serum ALT and AST levels were also frequently decreased [9,12,13,14,19,23,29]. Together, these findings indicate attenuation of hepatic steatosis and injury. Several molecular mechanisms observed in the liver may contribute to the microbiota-mediated metabolic effects of fungal interventions. Modulation of the hepatic AhR/TSC2/mTORC1 pathway was associated with improved insulin sensitivity [10]. Suppression of hepatic TLR4/JNK, TLR4/NF-κB/MAPK, and TLR4/MAPK signaling was linked to reduced hepatic inflammation and improvements in glucose and lipid metabolism [12,13,19]. Additional findings included decreased hepatic IL-6 and TNF-α levels [29] and normalization of the expression of genes involved in lipid metabolism, including decreased FABP1 and DGAT2 mRNA and increased Acsl3 mRNA [26].

In adipose tissue, the reported changes indicated enhanced thermogenesis and oxidative metabolism. Increased protein levels of UCP1 and key oxidative phosphorylation components were observed in brown and subcutaneous white adipose tissues [20]. mRNA expression of thermogenesis-related genes (*Ucp1*, *Dio2*, *Cidea*, *Ppara*, *Prdm16*, and *Ppargc1a*) was also increased in these tissues [20], while increased UCP1 mRNA expression in brown and inguinal white adipose tissues was reported in another study [26]. Consistently, stimulation of brown adipose tissue thermogenesis and browning of subcutaneous white adipose tissue were demonstrated [23]. These changes could increase energy expenditure and thus attenuate excessive adiposity. Decreased macrophage infiltration additionally suggested mitigation of adipose tissue inflammation [18]. Finally, increased protein levels of FGF21, PI3K, p-AKT, and GLUT4 in epididymal adipose tissue were in line with improved insulin sensitivity and enhanced glucose uptake [34].

Taken together, current evidence links gut microbiota changes to improved gut barrier integrity and reduced metabolic endotoxemia. These effects may, in turn, attenuate systemic, hepatic, and adipose tissue inflammation, improve insulin sensitivity and lipid metabolism. Further studies using pathway-specific antagonists and loss-of-function models are needed to determine whether the proposed mechanisms causally contribute to these effects. Until such evidence becomes available, the proposed mechanisms should be regarded as plausible rather than definitely established.

### 4.2. Potential Mediating Role of Microbiota-Associated Metabolites

Several studies included in this review examined whether microbiota-associated metabolites may contribute to the beneficial metabolic effects of fungal interventions. The available evidence primarily focuses on short-chain fatty acids (SCFAs) and tryptophan-derived metabolites, with one study additionally suggesting a role for brassicasterol. However, most proposed relationships are based on associations among microbial changes, metabolite concentrations, and host metabolic outcomes, whereas only a few studies combined microbiota manipulation with administration of the metabolite of interest.

Regarding SCFAs, more direct evidence was obtained for the possible involvement of butyrate in the protective activity of *Ganoderma lucidum* polysaccharides. Antibiotic treatment abolished both the anti-obesity effects of GLPs and the GLPs-associated increase in butyrate level. When administered alone, butyrate attenuated HFD-induced obesity by stimulating lipolysis and reducing adipose tissue inflammation, thereby reproducing several effects of GLPs. GLPs also promoted the expansion of *Lactobacillus johnsonii*. Administration of this bacterium suppressed HFD-induced obesity while increasing fecal butyrate. These findings support a possible GLPs–*Lactobacillus johnsonii*–butyrate axis, although they do not prove that *L. johnsonii* produced the butyrate or that butyrate was necessary for the effects of GLPs [18].

*Poria cocos* polysaccharide supplementation produced a range of beneficial metabolic effects and increased fecal SCFA concentrations. In the FMT experiment, the recipients of fecal material from polysaccharide-treated donors exhibited improvements in lipid metabolism and gut barrier function, together with increased colonic SCFA levels and modulation of the FGF21-PI3K/AKT signaling pathway. The transfer of the improved metabolic phenotype, accompanied by increased SCFA concentrations in the recipients, suggests their possible involvement, although the causal contribution of SCFAs was not established [34]. Similarly, *Auricularia auricula-judae* polysaccharides increased fecal concentrations of total SCFAs and several individual SCFAs, including acetic, isobutyric, and butyric acids. Improvements in the metabolic phenotype and increased concentrations of these total and individual SCFAs were observed in recipients after transfer of fecal material from polysaccharide-treated donors. These results support an association between SCFAs and the observed metabolic effects but do not demonstrate direct mediation [12].

Evidence concerning tryptophan-derived metabolites was reported for *Schizophyllum commune* and *Eurotium cristatum*. *Schizophyllum commune* polysaccharide alleviated HFD-induced obesity, enriched *Faecalibaculum rodentium*, and increased indole-3-lactic acid concentrations in feces and serum. The anti-obesogenic phenotype, enrichment of *F. rodentium*, and increased indole-3-lactic acid concentrations were also observed following FMT. The authors proposed that the effects of *S. commune* polysaccharide may involve modulation of the *Faecalibaculum rodentium*/indole-3-lactic acid/microRNA axis [29]. Crude extracellular polysaccharides produced by *E. cristatum* isolated from Fu brick tea ameliorated hyperglycemia and dyslipidemia in diabetic mice and increased colonic acetate and indole-3-propionic acid levels. Their metabolic effects were not present in pseudo-germ-free mice with T2DM. Further mechanistic investigation indicated that the beneficial metabolic effects may depend on remodeling of gut microbiota-dependent tryptophan metabolism and activation of the hepatic AhR/TSC2/mTORC1 axis [10].

The observation that *Cordyceps militaris* polysaccharide elevated hepatic levels of brassicasterol in a manner dependent on neomycin-sensitive gut bacteria suggested that this metabolite may participate in the demonstrated activity. Intraperitoneally administered brassicasterol reproduced the anti-obesity effects of the polysaccharide, including reduced body weight gain, improved lipid and glucose metabolism, and attenuated inflammation. Brassicasterol upregulated *Apoa4*, which may facilitate lipid transport and suppress inflammation both in vitro and in vivo. On this basis, the authors proposed a neomycin-sensitive gut bacteria–brassicasterol–Apoa4 pathway [15].

The studies included in this review provided evidence ranging from associations to more direct evidence from experiments involving antibiotic treatment, FMT, and administration of specific bacteria or pure metabolites. Further studies are needed to confirm the proposed pathways, establish causal mediation, and determine the microbial origin of the metabolites of interest.

### 4.3. Limitations of the Current Evidence and Future Research Needs

The studies selected for this review employed different approaches to examine whether the gut microbiota contributes causally to the metabolic effects of fungal interventions. Chaudhari et al. (2021) proposed a chain of evidence for microbiome-linked diseases that progresses from (1) association studies, through (2) observations in germ-free animals and antibiotic-treated animals or humans and (3) fecal microbiota transplantation, to (4) identification of specific strains and (5) molecules capable of eliciting a phenotype [36]. From this perspective, the included studies differ in the strength of the causal evidence they provide. Studies combining complementary approaches and progressing toward the experimental evaluation of specific microbial candidates or metabolites may therefore be considered particularly informative.

Several limitations should be considered. All causality-oriented experiments included in this review were conducted in rodents, predominantly mice. Confirmation in other translationally relevant animal models, such as pigs, would increase the translational value of the findings, while studies in humans are ultimately needed to establish their clinical relevance. In some of the FMT studies considered, recipients in the control group received vehicle alone. Inclusion of a control group receiving fecal material from disease-model donors not treated with the fungal intervention could allow the contribution of intervention-modulated microbiota to be assessed more reliably. Analysis of the microbiota in both donors and recipients would also help document the transfer and engraftment of donor-associated microbial features. However, such analyses were not consistently conducted. It should also be considered that transplanted fecal material may have contained undigested polysaccharides or other fungal-derived constituents that could affect recipients independently of the transferred microbiota. In some of the antibiotic-depletion experiments reviewed, the metabolic effects of antibiotic treatment were not evaluated by comparing disease-model control groups with and without antibiotic treatment, although such evaluation may be important because antibiotics themselves may influence metabolic outcomes. Finally, most studies reported relative rather than absolute taxon abundances. Because relative abundance may increase even when absolute abundance remains unchanged or decreases, and vice versa, complementary absolute quantification would improve interpretation. While a significantly lower Firmicutes/Bacteroidetes ratio was commonly reported, this pattern was not found in all included studies.

In FMT experiments, donors received fungal interventions over a relatively wide dose range, with most doses falling between 100 and 400 mg/kg/day, although some studies used lower or higher doses. Similarly, in antibiotic-depletion experiments, most doses of fungal interventions ranged from 200 to 400 mg/kg/day. When these animal doses are converted to human-equivalent doses using the body surface area normalization method, the calculated doses appear to be achievable in practice [37]. Nevertheless, such conversion does not establish efficacy or safety in humans and should be interpreted cautiously.

Fungal polysaccharides differed considerably in purity, monosaccharide composition, molecular weight, and the structures of their backbones and side chains, complicating comparisons across studies. Although they were the predominant interventions, information on their gastrointestinal fate was lacking. Additional data on their gastrointestinal stability, microbial degradation, and the resulting metabolites are therefore needed. Additionally, the extent of chemical characterization of the fungal interventions varied across studies and was limited in some cases. More comprehensive analyses would facilitate future cross-study comparisons and improve understanding of the relationship between chemical features and biological activity.

Further research is needed to clarify the causal contribution of the gut microbiota, identify the specific microorganisms and metabolites involved, and establish the molecular mechanisms underlying the metabolic benefits of fungal interventions.

## 5. Conclusions

Accumulating evidence suggests a possible causal role of the gut microbiota in mediating the positive metabolic effects of certain fungi and their polysaccharides. The studies addressing causality were specifically designed, employing FMT, antibiotic depletion experiments, cohousing, and treatments with bacterial strains previously shown to be enriched in the intervention group. The pharmacological effects observed in recipient mice and rats encompassed a wide range of benefits, including reduced body, visceral fat, and liver weights; lower serum levels of leptin, LPS, and proinflammatory cytokines; improved glycemic and lipid profiles; and enhanced insulin sensitivity. Histological findings revealed smaller adipocytes and attenuated hepatic steatosis. Upregulation of tight junction proteins in the colon suggests strengthened gut barrier function. Alterations in gut microbiota composition induced by fungi and their polysaccharides were diverse, with a decreased Firmicutes/Bacteroidetes ratio being the most frequently reported change. Oral administration of bacterial species enriched by these interventions, including *Papillibacter cinnamivorans*, *Parabacteroides goldsteinii*, *Bacteroides intestinalis*, *Lactobacillus johnsonii*, and *Enterococcus casseliflavus*, also produced metabolic benefits, suggesting their probiotic potential.

Overall, recent findings may open new perspectives for treating metabolic disorders through gut microbiota-targeting mushrooms and their polysaccharides. Further studies in this emerging field are strongly encouraged, particularly those designed to investigate causality and elucidate the underlying molecular mechanisms. Nevertheless, substantial work remains before these findings can be translated into clinical practice. The information summarized in this review should therefore be interpreted with caution, given the limitations of the animal models and experimental designs, as well as the inherent challenges of extrapolating preclinical evidence to human physiology.

## Figures and Tables

**Table 1:** Fungal interventions, animal models, associated gut microbiota changes, and outcomes relevant to metabolic health potentially linked to the gut microbiota.

#	Fungal Intervention and Animal Model	Gut Microbiota Changes Following Fungal Interventions, FMT, or Administration of Specific Bacterial Strains ^a^	Method(s) Used to Explore Causality and Outcomes Relevant to Metabolic Health Potentially Linked to the Gut Microbiota ^b^	Reference
1	fungal intervention purified water-soluble galactomannan (peak molecular weight (Mp): 26,827 Da; weight-average molecular weight (Mw): 32,305 Da; number-average molecular weight (Mn): 22,514 Da; monosaccharide composition: mainly mannose and galactose with small amounts of glucose) from mature spores of *Aspergillus cristatus* (syn.: *Eurotium cristatum*) isolated from Fuzhuan tea animal model HFD-induced obesity (C57BL/6J mice)	fungal intervention-treated mice alpha diversity metrics: ↑ Shannon index, Chao1 index phylum: ↑ Bacteroidetes ↓ Firmicutes ↓ Firmicutes/Bacteroidetes ratio genus: ↑ *Parabacteroides* ↓ *Lachnoclostridium* species: ↓ *Clostridium leptum*, *Mucispirillum schaedleri* ↑ *Parabacteroides goldsteinii*, *Parabacteroides distasonis*, *Faecalibaculum rodentium*, *Blautia producta*, *Bacteroides thetaiotaomicron*, *Bacteroides uniformis*	method to explore causality FMT outcomes relevant to metabolic health ↓ body weight, body weight gain ↓ liver weight and hepatic steatosis ↓ inguinal fat weight, epididymal fat weight ↓ adipocyte size ↓ fasting insulin levels and insulin resistance ↓ AUC (GTT) ↓ serum TG, serum TC, serum LDL ↑ serum HDL	Lu et al., 2025 [7]
2	fungal intervention live *Aspergillus cristatus* (syn.: *Eurotium cristatum*) isolated from Fuzhuan tea animal model HFD-induced obesity (C57BL/6J mice)	fungal intervention-treated mice phylum: ↓ Firmicutes ↑ Bacteroidetes genus: ↓ *Lactobacillus*, *Streptococcus*, *Anaerovibrio*, *Allobaculum* ↑ *Alistipes*, *Anaerotruncus*, *Oscillibacter*, *Helicobacter*, *Rikenella* species: ↓ *Lactobacillus reuteri* ↑ *Bacteroides acidifaciens*, *Blautia coccoides*, *Faecalibacterium prausnitzii* other taxa: ↓ Prevotellaceae UCG-003, Ruminococcus torques group ↑ Ruminococcaceae UCG-014, Lachnospiraceae NK4A136 group, unidentified Ruminococcaceae, Rikenellaceae RC9 gut group, Ruminococcaceae NK4A214 group, Ruminococcaceae UCG-010, Coprococcus 1, Ruminiclostridium 5	method to explore causality FMT outcomes relevant to metabolic health ↓ body weight, body weight gain ↓ inguinal fat, epididymal fat ↓ liver weight ↓ fasting glucose ↓ AUC (GTT) ↓ serum TG, serum TC, serum LDL	Lu et al., 2021 [8]
3	fungal intervention purified polysaccharides (content: 39.54% carbohydrate, 17.23% uronic acid, 26.37% protein; average molecular weight: 21.16 kDa; ribose, glucose, galactose, and mannose in a molar ratio of 1:1.7:4.4:5.2) from sporoderm-broken cleistothecia of *Aspergillus cristatus* (syn.: *Eurotium cristatum*) originating from Fu-brick tea animal model HFD-induced obesity (Sprague-Dawley rats)	fungal intervention-treated rats alpha diversity metrics: ↑ Shannon index ↓ Simpson index phylum: ↓ Firmicutes ↑ Bacteroidetes ↓ Firmicutes/Bacteroidetes ratio genus: ↓ *Blautia*, *Desulfovibrio*, *Lachnoclostridium*, *Roseburia*, *Streptococcus* ↑ *Bacteroides*, *Romboutsia*, *Akkermansia*, *Faecalibaculum*, *Parabacteroides* species: ↑ *Akkermansia muciniphila* other taxa: ↑ Ruminococcaceae_UCG-005, Clostridium_sensu_stricto_1 FMT recipient rats alpha diversity metrics: ↑ Shannon index ↓ Simpson index phylum: ↓ Firmicutes ↑ Bacteroidetes ↓ Firmicutes/Bacteroidetes ratio genus: ↑ *Akkermansia*, *Alloprevotella*, *Bacteroides*, *Faecalibaculum*, *Romboutsia*, *Rikenella*, *Roseburia* ↓ *Allobaculum*, *Blautia*, *Desulfovibrio*, *Streptococcus* other taxa: ↓ Escherichia-Shigella	method to explore causality FMT outcomes relevant to metabolic health ↓ body weight, body weight gain ↓ epididymal fat weight, perirenal fat weight ↓ epididymal adipocyte hypertrophy ↓ liver/body weight ratio, hepatic steatosis ↓ serum AST, serum ALT ↓ HOMA-IR ↓ serum TC, serum LDL, serum TG ↑ serum HDL ↑ periodic acid-Schiff-positive area in colon ↑ goblet cell count in colon ↑ colonic expression of tight junction proteins (occludin, zonula occludens-1, claudin-1) ↓ serum MCP-1, serum TNF-α, serum IL-6 ↓ serum LPS ↑ serum IL-10	Zhu et al., 2024 [9]
4	fungal intervention crude extracellular polysaccharides (purity: 47.45%; uronic acids: approximately 12.22%; proteins: approximately 10.80%; main monosaccharides: glucose, 70.53%, and galactose, 10.15%) produced by *Aspergillus cristatus* (syn.: *Eurotium cristatum*) originating from Fu brick tea animal model HFD- and STZ-induced T2DM (C57BL/6J mice)	fungal intervention-treated mice other taxa: ↓ norank_f__Eubacterium_coprostanoligenes_group	method to explore causality antibiotic treatment outcomes relevant to metabolic health Beneficial effects were attenuated under antibiotic treatment, as evidenced by no significant changes in the following parameters: AUC (GTT), AUC (ITT), fasting insulin, insulin sensitivity index, serum TC, serum TG, serum HDL, serum LDL, serum glucagon-like peptide-1, hepatic mRNA *Ahr*, hepatic mRNA *Tsc2*, hepatic mTORC1 content	Tan et al., 2026 [10]
5	fungal intervention *Auricularia auricula-judae* (syn.: *Auricularia auricula*) polysaccharides (content of polysaccharides: 97.6%; average molecular weight: 1065.2 kDa; mainly composed of mannose, glucose, xylose, galactose, glucuronic acid and fucose in a molar ratio of 50.84%, 21.61%, 9.24%, 8.58%, 5.78%, and 3.96%) animal model HFD-induced obesity (C57BL/6 mice)	fungal intervention-treated mice genus: ↓ *Mucispirillum* ↑ *Peptococcus*, *Muribaculum*, *Anaerovorax*, *Papillibacter*	methods to explore causality (1) antibiotic treatment, (2) FMT, (3) *Papillibacter cinnamivorans* treatment outcomes relevant to metabolic health (1) antibiotic treatment Beneficial effects were attenuated under antibiotic treatment, as evidenced by no significant changes in the following parameters: body weight, body weight gain, liver weight, abdominal fat pad weight, mRNA expression of adipogenesis and lipogenesis-related genes in abdominal fat, average area of fat cells, liver TG, plasma TG, plasma TC, plasma LDL, glycemia in GTT, AUC in GTT (2) FMT ↓ body weight, body weight gain ↓ glycemia and AUC (GTT) ↓ liver and plasma TG ↓ serum TC, serum LDL ↑ serum HDL ↓ abdominal fat pad, average area of fat cells ↓ mRNA expression of adipogenesis-related genes in abdominal fat (*Cebpα*, *Srebf1*, *Pparγ*, *Fasn*, *Acc1*, *Atgl*, *Lpl*) (3) *Papillibacter cinnamivorans* treatment ↓ body weight, relative weight gain ↓ liver and abdominal fat weight ↓ average area of fat cells ↓ mRNA expression of fat-metabolism-related genes in abdominal fat (*Cebpα*, *Acc1*) ↓ blood Glu and AUC (GTT) ↓ histological evidence of HFD-induced colon inflammation ↓ colon IL-6, colon TNF-α ↓ serum IL-6, serum TNF-α ↓ IL-6 mRNA, TNF-α mRNA, IL-18 mRNA in colon ↑ IL-17α mRNA, IL-22 mRNA in colon ↓ liver and serum TG ↓ serum cholesterol ↓ activation of JAK-STAT signaling ↑ protein and mRNA expression of zonula occludens-1 ↓ serum LPS ↓ intestinal lipid absorption ↑ hepatic thermogenesis	Zong et al., 2023 [11]
6	fungal intervention *Auricularia auricula-judae* polysaccharides (neutral sugar content: 67.68%; uronic acid content: 25.50%; main units: mannose and galacturonic acid; average molecular weight: 1210 kDa) animal model HFD-induced obesity (C57BL/6J mice)	fungal intervention-treated mice alpha diversity metrics: ↑ Simpson index phylum: ↑ Firmicutes, Bacteroidetes ↓ Proteobacteria FMT recipient mice alpha diversity metrics: ↑ Observed species, Simpson index phylum: ↑ Firmicutes, Bacteroidetes ↓ Proteobacteria ↓ Firmicutes/Bacteroidetes ratio genus: ↑ *Allobaculum*, *Roseburia*, *Anaerotruncus*, *Lactococcus*, *Alistipes* ↓ *Shigella*	method to explore causality FMT outcomes relevant to metabolic health ↓ body weight, liver weight ↓ Lee’s index ↓ epididymal adipose tissue weight/body weight ↓ mesenteric adipose tissue weight/body weight ↓ perirenal adipose tissue weight/body weight ↓ mean adipocyte size ↓ blood Glu ↓ AUC (GTT) ↑ fasting serum insulin ↓ HOMA-IR ↓ serum TC, serum TG, serum LDL, serum HDL ↓ LDL/HDL ratio ↓ serum AST, serum ALT ↑ intestinal barrier integrity (histological evidence) ↓ serum LPS, serum TNF-α, serum IL-6 ↓ TLR4/JNK signaling	Zhou et al., 2023 [12]
7	fungal intervention *Boletus edulis* purified polysaccharide (molecular weight: 11,485 Da; monosaccharide constituents: mannose and glucose) animal model HFD-induced obesity (ICR mice)	fungal intervention-treated mice alpha diversity and coverage metrics: ↑ Chao1 index, Simpson index, Shannon index, observed_species index, Pielou’s index ↓ Good_coverage index phylum: ↓ Firmicutes ↓ Firmicutes/Bacteroidetes ratio family: ↑ Lachnospiraceae, Lactobacillaceae, S24–7 ↓ Erysipelotrichaceae, Desulfovibrionaceae genus: ↑ [*Prevotella*], *Lactobacillus* ↓ *Allobaculum*, *Desulfovibrio* FMT recipient mice alpha diversity metrics: ↑ Chao1 index, Shannon index, observed_species index, Pielou-e index phylum: ↓ Firmicutes ↑ Bacteroidetes family: ↑ Lactobacillaceae, S24–7 ↓ Desulfovibrionaceae genus: ↑ *Lactobacillus*, *Bacteroides* ↓ *Allobaculum*, *Desulfovibrio*	method to explore causality FMT outcomes relevant to metabolic health ↓ body weight ↓ fasting blood glucose ↓ glycemia and AUC (GTT) ↓ serum TC, serum TG, serum LDL ↓ liver TC, liver TG, liver LDL ↓ serum ALT, serum AST ↓ adipose deposition ↓ serum LPS ↓ serum TNF-α ↑ mucin 2 and occludin in colon ↓ TLR4, Myd88, p-JNK/JNK, p-P38/P38, PEPCK, FOXO1, FASN, SREBP-1c in liver ↑ p-Akt/Akt in liver	Zhao et al., 2025 [13]
8	fungal intervention *Cordyceps militaris* neutral refined polysaccharide (molecular weight: 87.8 kDa; α- and β-glycosidic linkages; consists of mannose, galactose and glucose in a molar ratio of 2.2:15.1:1) animal model HFD- and STZ-induced T2DM (C57BL/6 mice)	fungal intervention-treated mice alpha diversity metrics: ↑ Sobs index, ACE index, Chao1 index, Shannon index ↓ Simpson index phylum: ↑ Bacteroidota, Campilobacterota ↓ Firmicutes/Bacteroidetes ratio genus: ↑ *Alistipes*, *Helicobacter* ↓ *Enterococcus* other taxa: ↑ norank_f_Muribaculaceae, Lachnospiraceae_NK4A136_group, norank_o_Clostridia_UCG-014, Eubacterium_xylanophilum_group ↓ Escherichia-Shigella	method to explore causality FMT outcomes relevant to metabolic health ↓ drinking water, food intake ↓ fasting blood Glu level ↓ AUC (GTT) ↓ fasting serum insulin level ↓ HOMA-IR ↓ serum TC, serum TG ↓ serum AST, serum ALT ↓ blood urea nitrogen level ↓ serum creatinine ↓ serum LPS ↓ TNF-α, IL-1β, IL-6, p-IκBα/IκBα, p-p65/p65, and TLR4 in colon ↑ claudin-1, occludin, and zonula occludens-1 in colon ↓ abnormal changes in histopathology of the pancreas and colon	Zhao et al., 2023 [14]
9	fungal intervention *Cordyceps militaris* polysaccharide (monosaccharide composition: 45.72% mannose, 24.78% galactose, and 29.50% glucose; molecular weight exceeds 670 kDa; structural backbone: linear (1 → 4)-linked α-D-glucopyranose) animal model HFD-induced obesity (C57BL/6J mice)	fungal intervention-treated mice phylum: ↑ Bacteroidetes ↓ Firmicutes genus: ↑ *Parabacteroides*, *Lactobacillus* species: ↑ *Parabacteroides goldsteinii*	method to explore causality antibiotic treatment outcomes relevant to metabolic health Beneficial effects were attenuated under neomycin treatment, as evidenced by no significant changes in the following parameters: body weight, body weight gain, energy intake, energy efficiency, epididymal fat weight, hepatic steatosis and inflammation (histological evidence), serum TC, serum TG, serum LDL, blood Glu level, serum insulin level, colon length, intestinal barrier injury (histological evidence), colon zonula occludens-1 and occludin protein levels, serum LPS	Cai et al., 2026 [15]
10	fungal intervention *Ganoderma lucidum* mycelium water extract animal model HFD-induced obesity (C57BL/6NCrlBltw mice)	fungal intervention-treated mice phylum: ↓ Firmicutes/Bacteroidetes ratio FMT recipient mice phylum: ↓ Proteobacteria ↓ Firmicutes/Bacteroidetes ratio species: ↓ *Mucispirillum schaedleri*, *Escherichia fergusonii*, *Lactococcus lactis*, *Clostridium scindens*, *Oscillibacter valericigenes*, *Clostridium cocleatum*, *Bacteroides caccae*, *Eubacterium dolichum*, *Coprobacillus cateniformis* ↑ *Parabacteroides goldsteinii*	method to explore causality FMT outcomes relevant to metabolic health ↓ body weight, liver weight ↓ epididymal fat, subcutaneous fat ↓ TNF-α mRNA, IL-1β mRNA, IL-6 mRNA, and MCP-1 mRNA in liver and adipose tissue ↑ occludin mRNA and zonula occludens-1 mRNA in the ileum ↓ lipogenic gene expression (ACC-1 mRNA, FAS mRNA, SREBP-1c mRNA, PPAR-γ mRNA in liver and adipose tissue)	Chang et al., 2015 [16]
11	fungal intervention *Ganoderma lucidum* polysaccharides from sporoderm-broken spores (total carbohydrate content: 80.87%; weight-average molecular weight: 26.0 kDa; composed of glucose, mannose, and galactose in a molar ratio of 87.4:4.81:8.14; β-D-glucan containing (1 → 3)-β-D-Glc*p*, (1 → 3,6)-β-D-Glc*p*, (1 → 6)-β-D-Glc*p*, and terminal-β-D-Glc*p* moieties) animal model HFD-induced obesity (C57BL/6J mice)	fungal intervention-treated mice species: ↑ *Bacteroides_chinchillae* other taxa: ↑ Christensenellaceae_R-7_group ↓ Ruminococcaceae_UCG-009, Lachnospiraceae_FCS020_group, Lachnospiraceae_UCG-001, Firmicutes_bacterium_ASF500	method to explore causality FMT outcomes relevant to metabolic health ↓ body weight, body weight gain ↓ epididymal white adipose tissue weight ↓ inguinal white adipose tissue weight ↓ serum LPS ↓ serum TNF-α	Sang et al., 2021 [17]
12	fungal intervention *Ganoderma lucidum* polysaccharides from sporoderm-broken spores (purity: 80.87%; average molecular weight: 26.0 kDa; monosaccharide composition: glucose, mannose, and galactose in a molar ratio of 87.04:4.81:8.14; four β-D-Glcp units: (1 → 3)-linked, (1 → 3,6)-linked, (1 → 6)-linked, and terminal) animal model HFD-induced obesity (C57BL/6J mice)	fungal intervention-treated mice alpha diversity metrics: ↓ Shannon index, Sobs index family: ↑ *Lactobacillaceae*, *Bifidobacteriaceae* ↓ *Oscillospiraceae* genus: ↓ *Colidextribacter*, *Roseburia*, *Dubosiella* ↑ *Lactobacillus*, *Bifidobacterium* species: ↑ *Lactobacillus johnsonii*, *Lactobacillus reuteri* other taxa: ↓ norank_f__Desulfovibrionaceae	methods to explore causality (1) antibiotic treatment, (2) *Lactobacillus johnsonii* treatment outcomes relevant to metabolic health (1) antibiotic treatment Beneficial effects were attenuated under antibiotic treatment, as evidenced by no significant changes in the following parameters: body weight, body weight gain, weight of white adipose tissue, AUC (GTT), cell diameter of adipocytes, macrophage infiltration in adipose tissue (F4/80-positive area), number of goblet cells per villus in the ileum, serum TG, serum LPS-binding protein, ileal FABP4 and PPARγ protein expression (2) *Lactobacillus johnsonii* treatment ↓ body weight gain ↓ weight of white adipose tissue ↓ adipocyte diameter in white adipose tissue ↓ fasting blood Glu, AUC (GTT) ↑ protein expression of adipose triglyceride lipase ↓ IL-6 and CCL2 mRNA levels in white adipose tissue ↓ macrophage infiltration in white adipose tissue (F4/80-positive area) ↑ number of goblet cells per villus of the ileum ↑ ileal claudin-1 and occludin immunofluorescence ↑ PPARγ protein expression in the ileum	Sang et al., 2026 [18]
13	fungal intervention purified uniform fraction of enzyme-hydrolyzed polysaccharides from *Ganoderma lucidum* fruiting bodies (sugar content: 93.87%; protein content: 0.94%; monosaccharide composition: fucose, galactose, glucose, and mannose in a molar ratio of 1.00:1.70:8.41:2.15; molecular weight: 33.602 kDa; molecular weight/number-average molecular weight ratio: 1.119) animal model HFD-induced NAFLD (C57BL/6J mice)	fungal intervention-treated mice alpha diversity metrics: ↑ Sobs index, ACE index, Chao index, Shannon index phylum: ↑ Patescibacteria ↓ Verrucomicrobiota, Proteobacteria genus: ↑ *Lachnoclostridium*, *Lactobacillus* ↓ *Akkermansia*, *Blautia*, *Romboutsia*, *Odoribacter* other taxa: ↑ Lachnospiraceae_NK4A136_group, norank_f__norank_o__Clostridia_UCG-014, norank_f__Muribaculaceae ↓ Escherichia-Shigella	method to explore causality FMT outcomes relevant to metabolic health ↓ serum ALT, serum AST ↓ serum LPS ↓ liver lipid accumulation ↓ TLR4/NF-κB/MAPK pathway in the liver	Zhao et al., 2026 [19]
14	fungal intervention *Inonotus obliquus* combined extract obtained by three successive hot-reflux extractions using 90% and 100% ethanol animal model HFD-induced obesity (C57BL/6N mice)	fungal intervention-treated mice genus: ↑ *Enterococcus* species: ↑ *Enterococcus casseliflavus*	methods to explore causality (1) FMT, (2) *Enterococcus casseliflavus* treatment outcomes relevant to metabolic health (1) FMT ↓ body weight ↓ liver weight ↓ brown, subcutaneous white, and epididymal white adipose tissue weights ↓ lipid droplet content in brown, subcutaneous white, and epididymal white adipose tissues ↑ rectal temperature in recipient mice during cold challenge ↓ adipocyte hypertrophy and hepatic steatosis ↑ protein levels of UCP1 and key oxidative phosphorylation subunits in brown and subcutaneous white adipose tissues ↑ mRNA expression of the thermogenic genes (*Ucp1*, *Dio2*, *Cidea*, *Ppara*, *Prdm16*, and *Ppargc1a*) in brown and subcutaneous white adipose tissues (2) *Enterococcus casseliflavus* treatment ↓ body weight ↓ liver weight and hepatic steatosis ↓ brown, subcutaneous white, and epididymal white adipose tissue weights ↓ lipid droplet content in brown, subcutaneous white, and epididymal white adipose tissues ↓ AUC (GTT and ITT) ↓ serum TC, serum TG, serum LDL ↑ serum HDL ↑ protein levels of UCP1 and key oxidative phosphorylation components in brown and subcutaneous white adipose tissues ↑ mRNA expression of the thermogenic genes (*Ucp1*, *Dio2*, *Cidea*, *Ppara*, *Prdm16*, and *Ppargc1a*) in brown and subcutaneous white adipose tissues ↑ volume of consumed oxygen ↑ mouse core body temperature after acute cold exposure ↑ number of colonic goblet cells ↓ inflammatory cell infiltration in colon ↓ histological score (colon tissue) ↑ intestinal tight junction proteins (zonula occludens-1, occludin, and claudin-1) ↓ TNF-α, IL-1β, IL-6, and MCP-1 mRNA ↓ serum LPS	Sun et al., 2026 [20]
15	fungal intervention *Isaria cicadae* (syn.: *Cordyceps cicadae*) crude polysaccharides animal model HFD- and STZ-induced T2DM (C57/BL6J mice)	fungal intervention-treated mice alpha diversity metrics: ↑ Chao index, Shannon index phylum: ↓ Firmicutes ↑ Bacteroidetes ↓ Firmicutes/Bacteroidetes ratio order: ↓ Clostridiales family: ↓ Lachnospiraceae genus: ↓ *Helicobacter*, *Lactobacillus*, *Ruminococcus* ↑ *Bacteroides*, *Odoribacter*, *Alloprevotella*, *Parabacteroides*, *Mucispirillum*, *Marvinbryantia* species: ↓ *Streptococcus hyointestinalis* ↑ *Bacteroides vulgatus*	method to explore causality FMT outcomes relevant to metabolic health ↓ water intake, urinary output ↓ blood Glu ↓ AUC (GTT) ↑ HOMA-IS	Wang et al., 2023 [21]
16	fungal intervention crude polysaccharides from fruiting bodies of *Lactifluus volemus* (syn.: *Lactarius volemus*) (polysaccharide content: 86.45%; protein content: 7.26%; monosaccharide composition: mannose, glucose, galactose, arabinose and fucose in a molar ratio of 4.14:1.0:11.67:7.89:2.35) animal model HFHFr diet-induced obesity (BALB/c mice)	fungal intervention-treated mice alpha diversity metrics: ↑ Chao1 index, Observed species index, Shannon index phylum: ↓ Firmicutes, Proteobacteria ↑ Bacteroidetes, Actinobacteria, Verrucomicrobia ↓ Firmicutes/Bacteroidetes ratio genus: ↑ *Bacteroides*, *Akkermansia*, *Bifidobacterium*, *Lactobacillus*, *Clostridium*, *Roseburia*, *Oscillospira* ↓ *Parasutterella*	method to explore causality antibiotic treatment outcomes relevant to metabolic health Beneficial effects were attenuated under antibiotic treatment, as evidenced by no significant changes in the following parameters: food efficiency ratio, adipose tissue weight, epididymal white adipose tissue weight, serum HDL, serum LDL, serum TC, serum TG, serum atherogenic index, fasting blood glucose, fasting serum insulin, HOMA-IR, insulin sensitivity index, AUC (GTT)	Xu et al., 2025 [22]
17	fungal intervention *Lyophyllum decastes* polysaccharides composed of polysaccharide 1 (number-average molecular weight Mn: 3.92 × 10^4^ Da; peak molecular weight Mp: 2.05 × 10^4^ Da; weight-average molecular weight Mw: 5.02 × 10^5^ Da; Z-average molecular weight Mz: 1.85 × 10^6^ Da; monosaccharide composition: mannose/glucose/galactose/fucose = 1:2.38:2.58:0.73; protein content: 0.12%) and polysaccharide 2 (number-average molecular weight Mn: 4.03 × 10^5^ Da; peak molecular weight Mp: 1.17 × 10^6^ Da; weight-average molecular weight Mw: 1.13 × 10^6^ Da; Z-average molecular weight Mz: 1.77 × 10^6^ Da; monosaccharide composition: mannose/glucose/galactose/fucose = 1:2.33:2.51:0.78; protein content: 2.40%) animal model HFD-induced obesity (C57BL/6J mice)	fungal intervention-treated mice phylum: ↑ Bacteroidetes family: ↑ Bacteroidaceae genus: ↑ *Bacteroides* species: ↑ *Bacteroides intestinalis*, *Lactobacillus johnsonii* bacterial intervention-treated mice (*Bi* = *Bacteroides intestinalis*, *Lj* = *Lactobacillus johnsonii*, *Bi+Lj* = *Bacteroides intestinalis* + *Lactobacillus johnsonii*) alpha diversity metrics: ↑ Simpson index (*Lj*), Chao1 index (*Lj*, *Bi+Lj*), Shannon index (*Lj*, *Bi+Lj*) phylum: ↑ Bacteroidetes (*Bi*, *Lj*), Verrucomicrobiota (*Bi*, *Lj*, *Bi+Lj*) ↓ Firmicutes/Bacteroidetes ratio (*Bi*, *Lj*) family: ↑ Bacteroidaceae (*Bi*), Akkermansiaceae (*Bi*, *Lj*, *Bi+Lj*) genus: ↑ *Bacteroides* (*Bi*), *Parabacteroides* (*Bi*), *Akkermansia* (*Bi*, *Lj*, *Bi+Lj*) species: ↑ *Bacteroides vulgatus* (*Bi*), *Bacteroides sartorii* (*Bi*), *Bacteroides acidifaciens* (*Bi*), *Parabacteroides distasonis* (*Bi*), *Lactobacillus johnsonii* (*Lj*, *Bi+Lj*), *Akkermansia muciniphila* (*Bi*, *Lj*, *Bi+Lj*) ↓ *Parabacteroides distasonis* (*Bi+Lj*)	method to explore causality oral treatment with *Bacteroides intestinalis* (*Bi*), *Lactobacillus johnsonii* (*Lj*), or a combination of these two strains (*Bi+Lj*) outcomes relevant to metabolic health ↓ body weight (*Bi*, *Lj*, *Bi+Lj*) ↓ total white adipose tissue weight (*Bi*, *Lj*, *Bi+Lj*) ↓ subcutaneous white adipose tissue weight (*Bi*, *Lj*, *Bi+Lj*) ↓ mesenteric white adipose tissue weight (*Bi*, *Lj*, *Bi+Lj*) ↓ relative cell diameter of adipocytes (*Bi*, *Lj*, *Bi+Lj*) ↓ plasma TC (*Bi*, *Lj*, *Bi+Lj*) ↓ plasma TG (*Lj*) ↓ plasma LDL (*Bi*, *Bi+Lj*) ↓ hepatic TC (*Bi*, *Lj*, *Bi+Lj*) ↓ hepatic TG, hepatic LDL (*Bi*, *Bi+Lj*) ↓ plasma AST, plasma ALT (*Bi*, *Lj*, *Bi+Lj*) ↑ brown adipose tissue thermogenesis (*Bi*, *Lj*, *Bi+Lj*) ↑ browning of subcutaneous white adipose tissue (*Bi*, *Lj*, *Bi+Lj*)	Wang et al., 2022 [23]
18	fungal intervention [24,25] *Morchella esculenta* water-soluble polysaccharides animal model [24,25] HFD-induced obesity (C57BL/6J mice)	fungal intervention-treated mice [24] alpha diversity metrics: ↑ Shannon index phylum: ↓ Firmicutes/Bacteroidetes ratio genus: ↑ *Lactobacillus*, *Dubosiella* ↓ *Faecalibaculum* other taxa: ↑ Rikenellaceae RC9	method to explore causality [25] antibiotic treatment outcomes relevant to metabolic health [25] Beneficial effects shown in a study by Liu et al. (2023) [24] were attenuated under antibiotic treatment, as evidenced by no significant changes in the following parameters: weight gain rate, AUC in GTT, epididymal fat index, liver index, serum AST, serum ALT	Liu et al., 2023; Zang et al., 2025 [24,25]
19	fungal intervention *Hirsutella sinensis* (anamorph of *Ophiocordyceps sinensis*) mycelium water extract (HSM) and its fraction H1 containing high-molecular weight polysaccharides (molecular weight: >300 kDa; monosaccharide composition of H1: mannose, glucose, galactose, *N*-glucosamine, arabinose, *N*-galactosamine, rhamnose, fucose in a ratio of 50.4%:12.5%:23.8%:4.6%:1.7%:0.4%:3.4%:3.2%) animal model HFD-induced obesity (C57BL/6J mice)	H1-treated mice species: ↑ *Parabacteroides goldsteinii*, *Ruminococcus gnavus*, *Intestinimonas butyriciproducens*, *Clostridium cocleatum*, *Ruminococcus flavefaciens*, *Butyrivibrio hungatei*, *Ochrobactrum anthropi*, *Ruminococcus bromii*, *Achromobacter insolitus*, *Delftia acidovorans*, *Haemophilus haemolyticus* ↓ *Bacteroides acidifaciens*, *Mucispirillum schaedleri*, *Dorea longicatena*, *Romboutsia timonensis*, *Shewanella algae*	methods to explore causality (1) antibiotic treatment, (2) FMT, (3) FMT+ex vivo antibiotic treatment of feces, (4) *Parabacteroides goldsteinii* treatment outcomes relevant to metabolic health (1) antibiotic treatment (H1) Beneficial effects of H1 were attenuated under treatment with neomycin or an antibiotic cocktail containing neomycin, as evidenced by no significant changes in the following parameters: body weight gain, visceral fat pad weight, HOMA-IR index, serum TNF-α, serum endotoxin, intestinal permeability, crown-like structures/100 adipocytes, NASH activity index score (2) FMT (HSM, H1) ↓ body weight gain, visceral fat pad ↓ HOMA-IR ↓ serum IL-1β, serum TNF-α ↓ serum endotoxin ↑ colonic zonula occludens-1 mRNA ↓ intestinal permeability (FITC-dextran) ↓ adipocyte mean size ↓ crown-like structures/100 adipocytes ↓ NASH activity index score (3) FMT + ex vivo antibiotic treatment of feces (H1) Ex vivo treatment of fecal microbiota from H1-treated donors with neomycin or a neomycin-containing antibiotic cocktail abolished the beneficial effects of FMT on body weight gain, visceral fat pad weight, HOMA-IR index, serum IL-1β, serum TNF-α, serum endotoxin, and intestinal permeability (4) *Parabacteroides goldsteinii* treatment ↓ body weight gain, visceral fat pad ↓ HOMA-IR ↓ serum IL-1β ↓ serum endotoxin ↓ intestinal permeability (FITC-dextran) ↓ IL-1β mRNA in colon ↑ IL-10 mRNA in colon ↑ zonula occludens-1 mRNA in colon ↓ adipocyte mean size ↓ crown-like structures/100 adipocytes ↑ UCP1 mRNA in brown adipose tissues and inguinal white adipose tissues ↓ NASH activity index score normalizes gene expression involved in lipid metabolism in the liver (↓ FABP1 mRNA, DGAT2 mRNA; ↑ Acsl3 mRNA)	Wu et al., 2019 [26]
20	fungal intervention *Phellinus igniarius* polysaccharides animal model HFD- and STZ-induced T2DM (C57/BL6J mice)	fungal intervention-treated mice alpha diversity metrics: ↑ Chao1 index genus: ↑ *Bacteroides*, *Lactobacillus*, *Alloprevotella*, *Alistipes*, *Parabacteroides* ↓ *Helicobacter*, *Desulfovibrio*, *Odoribacter*, *Mucispirillum*, *Ruminiclostridium*	method to explore causality FMT outcomes relevant to metabolic health ↓ water intake ↓ blood glucose, AUC (GTT), insulin ↓ IL-6, IL-1β, TNF-α in blood ↓ urinary output ↓ abnormal islet morphology ↑ clear islet boundaries ↓ swelling of the liver and kidney ↓ fatty tissue surrounding the kidney ↓ vacuolization and swelling of hepatocytes ↓ glomerular volume hypertrophy and renal mesangial hyperplasia	Ni et al., 2023 [27]
21	fungal intervention *Ramaria botrytoides* purified polysaccharide (total carbohydrate content: 89.33%; uronic acid content: 4.14%; protein content: 3.29%; average molecular weight: 3.635 kDa; monosaccharide composition: fucose, galactose, glucose, and mannose in a molar ratio of 0.058:0.349:0.515:0.079) animal model HFD- and STZ-induced T2DM (C57BL/6J mice)	fungal intervention-treated mice alpha diversity metrics: ↑ Chao1 index phylum: ↓ Firmicutes, Proteobacteria, Cyanobacteria ↑ Bacteroidetes, Actinobacteria, Verrucomicrobia, Tenericutes, Deferribacteres genus: ↑ *Alistipes*, *Bacteroides*, *Ruminococcus*, *Odoribacter*, *Akkermansia*, *Lactobacillus*, *Alloprevotella*, *Turicibacter*, *Ruminiclostridium* ↓ *Desulfovibrio*, *Enterorhabdus* other taxa: ↓ Candidatus_Saccharimonas	method to explore causality FMT outcomes relevant to metabolic health ↓ food and water intake ↓ urinary output ↓ liver lipid accumulation ↓ hepatocyte hypertrophy ↓ pancreatic islet structure damage ↓ blood glucose, AUC (GTT), insulin level	Ni et al., 2025 [28]
22	fungal intervention polysaccharide from *Schizophyllum commune* fruiting bodies (monosaccharide composition: fucose, glucosamine hydrochloride, galactose, glucose, and mannose in a relative molar ratio of 14:6:210:593:177; molecular weight: 15.1 kDa) animal model HFD-induced obesity (C57BL/6J mice)	fungal intervention-treated mice phylum: ↑ Bacteroidota species: ↓ *Bifidobacterium pseudolongum* ↑ *Faecalibaculum rodentium* FMT recipient mice species: ↓ *Romboutsia ilealis* ↑ *Faecalibaculum rodentium*, *Lactobacillus johnsonii*, *Lactobacillus reuteri*	method to explore causality FMT outcomes relevant to metabolic health ↓ body weight ↓ liver index ↓ epididymal white adipose tissue index ↓ number and size of lipid droplets in liver and epididymal white adipose tissue adipocytes ↓ serum TC, serum TG, serum LDL ↑ serum HDL ↓ serum non-esterified fatty acids ↓ serum LPS ↓ serum diamine oxidase ↑ liver catalase, liver total antioxidant capacity ↓ liver malondialdehyde ↓ liver TC, liver TG ↓ serum AST, serum ALT ↓ liver IL-6, liver TNF-α ↓ serum IL-6, serum TNF-α ↓ serum D-lactate ↑ ZO-1 mRNA ↑ length and number of colonic villi	Yin et al., 2026 [29]
23	fungal intervention *Sparassis latifolia* purified polysaccharides (purity: 95%; molecular weight: 215–393 kDa; structure: β-(1 → 3)-D-glucan main chain with β-(1 → 6)-D-glucan branches; monosaccharide composition: galactose, glucose, rhamnose, mannose, and fructose in a molar ratio of 10.55:24.76:2.51:5.64:1.3) animal model HFHS diet- and STZ-induced T2DM (C57BL/6 mice)	fungal intervention-treated mice phylum: ↓ Firmicutes/Bacteroidetes ratio	method to explore causality cohousing outcomes relevant to metabolic health ↓ fasting blood glucose ↓ blood glucose at 2 h (GTT), AUC (GTT) ↓ intestinal crypt depth	Wei et al., 2026 [30]
24	fungal intervention *Trametes versicolor* (syn.: *Coriolus versicolor*) protein-bound β-glucan (molecular weight: 53.9 kDa; monosaccharide composition: glucose; mainly composed of 4-linked and 4,6-linked glucosyl residues (77.4%), along with a small portion of 3-linked and 3,6-linked glucosyl residues (8.8%); peptide residues main amino acid: tyrosine) animal model HFD-induced obesity (C57Bl/6J mice)	fungal intervention-treated mice phylum: ↑ Verrucomicrobia genus: ↑ *Akkermansia*, *Bacteroides*, *Bacterium* other taxa: ↑ Ruminiclostridium_10	method to explore causality FMT outcomes relevant to metabolic health ↓ body weight ↑ serum adiponectin ↓ serum leptin ↓ serum IL-6, serum IL-1β, serum TNF-α	Li et al., 2019 [31]
25	fungal intervention *Tremella fuciformis* polysaccharide (molecular weight distribution: 10.07 to 66.29 kDa; main molecular weight: 37.28 kDa; composed of mannose, rhamnose, glucuronic acid, glucose, galactose, and xylose in a molar ratio of 15.95:0.31:2.47:1.20:3.18:1.72) animal model HFD-induced obesity (C57BL/6J mice)	fungal intervention-treated mice alpha diversity metrics: ↑ Simpson index phylum: ↓ Firmicutes ↑ Bacteroidota ↓ Firmicutes/Bacteroidota ratio family: ↓ Desulfovibrionaceae, Clostridiaceae ↑ Muribaculaceae genus: ↑ *Alistipes* ↓ *Desulfovibrio*, *Bilophila* FMT recipient mice phylum: ↓ Firmicutes ↑ Bacteroidota ↓ Firmicutes/Bacteroidota ratio family: ↑ Muribaculaceae, Peptococcaceae genus: ↑ *Blautia*, *Colidextribacter* other taxa: ↓ Clostridia_UCG-014 ↑ Lachnospiraceae_NK4A136_group	method to explore causality FMT outcomes relevant to metabolic health ↓ epididymal fat weight, perirenal fat weight ↓ liver weight ↓ adipocyte size ↓ fasting glucose ↓ serum TC, serum TG, serum LDL ↑ serum HDL ↑ serum glucagon-like peptide-1 ↑ serum glucagon-like peptide-2	He et al., 2022 [32]
26	fungal intervention water-insoluble polysaccharide from the sclerotium of *Wolfiporia cocos* (syn.: *Poria cocos*) (main chain: (1-3)-β-D-glucan; weight-average molecular weight (Mw): 4486 kDa; number-average molecular weight (Mn): 403.1 kDa; monosaccharide composition: glucose) animal model *ob*/*ob* mice	fungal intervention-treated mice alpha diversity metrics: ↓ Shannon index phylum: ↑ Bacteroidetes family: ↑ Lachnospiraceae genus: ↑ *Alloprevotella*, *Parabacteroides*, *Ruminococcus*, *Bacteroides* ↓ *Megamonas*, *Proteus* other taxa: ↑ Clostridium IV	method to explore causality FMT outcomes relevant to metabolic health ↓ weight change ↓ free-diet blood Glu ↓ plasma TC, plasma TG ↑ insulin sensitivity index	Sun et al., 2019 [33]
27	fungal intervention *Wolfiporia cocos* (syn.: *Poria cocos*) polysaccharides animal model HFD-induced obesity (C57BL/6J mice)	fungal intervention-treated mice alpha diversity metrics: ↑ Chao1 index, Shannon index genus: ↑ *Lactobacillus*, *Allobaculum*, *Phascolarctobacterium*	method to explore causality FMT outcomes relevant to metabolic health ↓ body weight ↓ fasting insulin, HOMA-IR ↓ serum TC, serum TG, serum LDL ↓ serum leptin ↓ leptin-to-adiponectin ratio ↑ number of adipocytes ↓ epididymal adipocyte area ↓ IL-6 mRNA, IL-1β mRNA, and TNF-α mRNA in epididymal adipose tissue ↓ IL-6 mRNA, IL-1β mRNA in colon ↑ claudin-1 in colon ↓ serum LPS ↑ protein expression levels of FGF21, PI3K, p-AKT, and GLUT4 in the epididymal adipose tissue	Liu et al., 2025 [34]
28	fungal intervention *Wolfiporia cocos* (syn.: *Poria cocos*) oligosaccharides (monosaccharide composition: mannose (0.57%), glucose (93.16%), galactose (3.49%), arabinose (2.78%); polymerization degree: 2–6; protein content: 1.3%; total sugar content: 94.0%) animal model HFD-induced obesity (C57BL/6J mice)	fungal intervention-treated mice phylum: ↑ Bacteroidetes; ↓ Firmicutes family: ↓ Ruminococcaceae, Anaeroplasmataceae ↑ Lactobacillaceae, Rikenellaceae genus: ↑ *Lactobacillus*, *Bacteroides* ↓ *Faecalibaculum*, *Helicobacter*, *Desulfovibrio*, *Mucispirillum*, *Alistipes* other taxa: ↑ Clostridium_sensu_stricto_1 ↓ Lachnospiraceae_NK4A136_group, Ruminococcus_1 FMT recipient mice alpha diversity metrics: ↑ Shannon index genus: ↑ *Bifidobacterium*, *Desulfovibrio*, *Bacteroides* ↓ *Staphylococcus*, *Roseburia* other taxa: ↓ Lachnospiraceae_NK4A136_group	methods to explore causality (1) antibiotic treatment, (2) FMT outcomes relevant to metabolic health (1) antibiotic treatment Beneficial effects were attenuated under antibiotic treatment, as evidenced by no significant changes in the following parameters: fasting blood glucose level, glycemia in intraperitoneal glucose tolerance test, glycemia and AUC in insulin tolerance test (2) FMT ↓ body weight ↓ pancreas weight	Zhu et al., 2022 [35]

^a^ The gut microbiota changes listed in this column were observed in disease-model animals after administration of a fungal intervention or specific bacterial strains, or in recipient animals after FMT using fecal microbiota from disease-model donors treated with the fungal intervention. The symbols ↓ and ↑ indicate lower and higher values, respectively, in animals receiving the respective intervention than in the corresponding controls. ^b^ In FMT experiments, the changes listed in this column were observed in recipient animals after transfer of fecal microbiota from disease-model donors treated with the fungal intervention, relative to the corresponding control groups. For cohousing and administration of specific bacterial strains, the changes were observed in disease-model intervention groups relative to the corresponding disease-model control groups. In cohousing experiments, disease-model animals were cohoused with disease-model animals treated with the fungal intervention. The symbols ↓ and ↑ indicate lower and higher values, respectively, relative to the corresponding controls. In antibiotic-depletion experiments, parameters are listed as unchanged only when the fungal intervention produced a beneficial change in disease-model animals relative to the corresponding disease-model controls in the absence of antibiotics, but the same beneficial change was no longer detected when the intervention was combined with antibiotic treatment. In the experiment combining FMT with ex vivo antibiotic treatment, the listed beneficial effects transferred by fecal microbiota from fungal intervention-treated donors were abolished when the donor fecal microbiota was treated with antibiotics before transplantation. Accordingly, all outcomes listed in this column were considered potentially linked to the gut microbiota. Abbreviations: ALT, alanine aminotransferase; AST, aspartate aminotransferase; AUC, area under the curve; FMT, fecal microbiota transplantation; Glu, glucose; GTT, glucose tolerance test; HDL, high-density lipoprotein; HFD, high-fat diet; HFHFr, high-fat/high-fructose; HFHS, high-fat/high-sugar; HOMA-IR, homeostasis model assessment of insulin resistance; ITT, insulin tolerance test; LDL, low-density lipoprotein; LPS, lipopolysaccharide; STZ, streptozotocin; T2DM, type 2 diabetes mellitus; TC, total cholesterol; TG, triglycerides; TNF-α, tumor necrosis factor-alpha.

## Data Availability

No new data were created or analyzed in this study.

## Supplementary Materials

The following supporting information can be downloaded at: https://www.mdpi.com/article/10.3390/nu18193281/s1, Figure S1: Study selection flow diagram; Table S1: Complete search strategies and search yields.
