# Supplementary material for: The Gut Microbiota as a Mediator of the Metabolic Benefits of Fungi and Their Polysaccharides: A Review of Evidence Addressing Causality
Source: Nutrients. 2026 Oct 6;18(19):3281. doi: 10.3390/nu18193281 (PMC13666899; doi:10.3390/nu18193281)
Supplement: Supplementary file 1 [file nutrients-18-03281-s001.zip › nutrients-4521886-supplementary.pdf]

SUPPLEMENTARY MATERIALS

Gut Microbiota as a Mediator of Metabolic Benefits of Fungi and Their Polysaccharides: A Review of Evidence Addressing Causality

Stevan Samardžić [1,*], Dragana D. Božić [2], Milica Drobac [1], Mirjana Marčetić [1], Jelena Arsenijević [1], Djordje Medarević [3] and Zoran Maksimović [1,4]

[1] Faculty of Pharmacy, Department of Pharmacognosy, University of Belgrade, Vojvode Stepe 450, 11221 Belgrade, Serbia
[2] Faculty of Pharmacy, Department of Microbiology and Immunology, University of Belgrade, Vojvode Stepe 450, 11221 Belgrade, Serbia
[3] Faculty of Pharmacy, Department of Pharmaceutical Technology and Cosmetology, University of Bel-grade, Vojvode Stepe 450, 11221 Belgrade, Serbia
[4] Planet Systems Group d.o.o., Sestara Ninković 5, 21000 Novi Sad, Serbia
* Correspondence: stevan.samardzic@pharmacy.bg.ac.rs; Tel.: +381-11-3951321

Table S1. Complete search strategies and search yields.

Database	Search date	Complete search strategy	Records retrieved
Scopus	1 August 2026	(TITLE-ABS-KEY(mushroom* OR fungus OR fungi OR macrofungus OR macrofungi OR microfungus OR microfungi OR macromycetes OR micromycetes OR mycelium OR polysaccharide* OR heteropolysaccharide* OR "beta-glucan*" OR "β-glucan*") AND TITLE-ABS-KEY("gut microbiota" OR "gut microbiome" OR "gut flora" OR "gut microflora" OR "intestinal microbiota" OR "intestinal microbiome" OR "intestinal flora" OR "intestinal microflora" OR "colonic microbiota" OR "colonic microbiome" OR "colonic flora" OR "colonic microflora" OR "fecal microbiota" OR "fecal microbiome" OR "fecal flora" OR "fecal microflora" OR "microbiota composition" OR "microbiome composition" OR "microflora composition" OR "flora composition") AND TITLE-ABS-KEY("metabolic syndrome" OR "metabolic disorder*" OR "metabolic dysfunction*" OR "metabolic disease*" OR obesity OR "body weight" OR adiposity OR "body mass" OR "body mass index" OR diabetes OR T2D OR prediabetes OR hyperinsulinemia OR "insulin resistance" OR "insulin sensitivity" OR "glucose tolerance" OR "glucose metabolism" OR "glucose homeostasis" OR hyperglycemia OR "non-alcoholic fatty liver disease" OR "nonalcoholic fatty liver disease" OR NAFLD OR "metabolic dysfunction-associated steatotic liver disease" OR MASLD OR "nonalcoholic steatohepatitis" OR "non-alcoholic steatohepatitis" OR NASH OR MASH OR "metabolic dysfunction-associated steatohepatitis" OR "fatty liver" OR "hepatic steatosis" OR "lipid metabolism" OR dyslipidemia OR hyperlipidemia OR hyperlipidaemia OR "energy metabolism" OR "energy expenditure") AND ALL("microbiota transplantation" OR "microbiome transplantation" OR "microflora transplantation" OR "flora transplantation" OR "fecal transplantation" OR FMT OR "microbiota transfer" OR "microbiome transfer" OR "fecal transfer" OR "microflora transfer" OR "flora transfer" OR "antibiotic-treated" OR "antibiotic pre-treatment" OR "antibiotic cocktail" OR "antibiotic depletion" OR "microbiota-depleted" OR	760

		"microbiome-depleted" OR "microflora-depleted" OR "flora-depleted" OR "germ-free" OR "gnotobiotic" OR "causal relationship" OR "causal role" OR causality OR "cause-effect" OR "cause and effect" OR "microbiota-mediated" OR "microbiome-mediated" OR "microflora-mediated" OR "flora-mediated" OR "microbiota-dependent" OR "microbiome-dependent" OR "microflora-dependent" OR "flora-dependent" OR cohous* OR "co-hous*" OR cohabit*)) AND (LIMIT-TO (DOCTYPE,"ar")) AND (LIMIT-TO (LANGUAGE,"English"))	
PubMed	1 August 2026	(((((mushroom*[Title/Abstract] OR fungus[Title/Abstract] OR fungi[Title/Abstract] OR macrofungus[Title/Abstract] OR macrofungi[Title/Abstract] OR microfungus[Title/Abstract] OR microfungi[Title/Abstract] OR macromycetes[Title/Abstract] OR micromycetes[Title/Abstract] OR mycelium[Title/Abstract] OR polysaccharide*[Title/Abstract] OR heteropolysaccharide*[Title/Abstract] OR "beta-glucan*"[Title/Abstract] OR "β-glucan*"[Title/Abstract]) AND ("gut microbiota"[Title/Abstract] OR "gut microbiome"[Title/Abstract] OR "gut flora"[Title/Abstract] OR "gut microflora"[Title/Abstract] OR "intestinal microbiota"[Title/Abstract] OR "intestinal microbiome"[Title/Abstract] OR "intestinal flora"[Title/Abstract] OR "intestinal microflora"[Title/Abstract] OR "colonic microbiota"[Title/Abstract] OR "colonic microbiome"[Title/Abstract] OR "colonic flora"[Title/Abstract] OR "colonic microflora"[Title/Abstract] OR "fecal microbiota"[Title/Abstract] OR "fecal microbiome"[Title/Abstract] OR "fecal flora"[Title/Abstract] OR "fecal microflora"[Title/Abstract] OR "microbiota composition"[Title/Abstract] OR "microbiome composition"[Title/Abstract] OR "microflora composition"[Title/Abstract] OR "flora composition"[Title/Abstract])) AND ("metabolic syndrome"[Title/Abstract] OR "metabolic disorder*"[Title/Abstract] OR "metabolic dysfunction*"[Title/Abstract] OR "metabolic disease*"[Title/Abstract] OR obesity[Title/Abstract] OR "body weight"[Title/Abstract] OR adiposity[Title/Abstract] OR "body mass"[Title/Abstract] OR "body mass index"[Title/Abstract] OR diabetes[Title/Abstract] OR T2D[Title/Abstract] OR prediabetes[Title/Abstract] OR hyperinsulinemia[Title/Abstract] OR "insulin resistance"[Title/Abstract] OR "insulin sensitivity"[Title/Abstract] OR "glucose tolerance"[Title/Abstract] OR "glucose metabolism"[Title/Abstract] OR "glucose homeostasis"[Title/Abstract] OR hyperglycemia[Title/Abstract] OR "non-alcoholic fatty liver disease"[Title/Abstract] OR "nonalcoholic fatty liver disease"[Title/Abstract] OR NAFLD[Title/Abstract] OR "metabolic dysfunction-associated steatotic liver disease"[Title/Abstract] OR MASLD[Title/Abstract] OR "nonalcoholic steatohepatitis"[Title/Abstract] OR "non-alcoholic steatohepatitis"[Title/Abstract] OR NASH[Title/Abstract] OR MASH[Title/Abstract] OR "metabolic dysfunction-associated steatohepatitis"[Title/Abstract] OR "fatty liver"[Title/Abstract] OR "hepatic steatosis"[Title/Abstract] OR "lipid metabolism"[Title/Abstract] OR dyslipidemia[Title/Abstract] OR hyperlipidemia[Title/Abstract] OR hyperlipidaemia[Title/Abstract] OR "energy metabolism"[Title/Abstract] OR "energy expenditure"[Title/Abstract])) AND ("microbiota transplantation" OR "microbiome transplantation" OR "microflora transplantation" OR "flora transplantation" OR "fecal transplantation" OR FMT OR "microbiota transfer" OR "microbiome transfer" OR "fecal transfer" OR "microflora transfer" OR "flora transfer" OR "antibiotic-treated" OR "antibiotic pre-treatment" OR "antibiotic cocktail" OR "antibiotic depletion" OR "microbiota-depleted" OR "microbiome-depleted" OR "microflora-depleted" OR	606

		"flora-depleted" OR "germ-free" OR "gnotobiotic" OR "causal relationship" OR "causal role" OR causality OR "cause-effect" OR "cause and effect" OR "microbiota-mediated" OR "microbiome-mediated" OR "microflora-mediated" OR "flora-mediated" OR "microbiota-dependent" OR "microbiome-dependent" OR "microflora-dependent" OR "flora-dependent" OR cohous* OR "co-hous*" OR cohabit*)) NOT (review[Title])) NOT (review[Publication Type]) Filters: English	
Web of Science	1 August 2026	mushroom* OR fungus OR fungi OR macrofungus OR macrofungi OR microfungus OR microfungi OR macromycetes OR micromycetes OR mycelium OR polysaccharide* OR heteropolysaccharide* OR "beta-glucan*" OR "β-glucan*" (Topic) and "gut microbiota" OR "gut microbiome" OR "gut flora" OR "gut microflora" OR "intestinal microbiota" OR "intestinal microbiome" OR "intestinal flora" OR "intestinal microflora" OR "colonic microbiota" OR "colonic microbiome" OR "colonic flora" OR "colonic microflora" OR "fecal microbiota" OR "fecal microbiome" OR "fecal flora" OR "fecal microflora" OR "microbiota composition" OR "microbiome composition" OR "microflora composition" OR "flora composition" (Topic) and "metabolic syndrome" OR "metabolic disorder*" OR "metabolic dysfunction*" OR "metabolic disease*" OR obesity OR "body weight" OR adiposity OR "body mass" OR "body mass index" OR diabetes OR T2D OR prediabetes OR hyperinsulinemia OR "insulin resistance" OR "insulin sensitivity" OR "glucose tolerance" OR "glucose metabolism" OR "glucose homeostasis" OR hyperglycemia OR "non-alcoholic fatty liver disease" OR "nonalcoholic fatty liver disease" OR NAFLD OR "metabolic dysfunction-associated steatotic liver disease" OR MASLD OR "nonalcoholic steatohepatitis" OR "non-alcoholic steatohepatitis" OR NASH OR MASH OR "metabolic dysfunction-associated steatohepatitis" OR "fatty liver" OR "hepatic steatosis" OR "lipid metabolism" OR dyslipidemia OR hyperlipidemia OR hyperlipidaemia OR "energy metabolism" OR "energy expenditure" (Topic) and "microbiota transplantation" OR "microbiome transplantation" OR "microflora transplantation" OR "flora transplantation" OR "fecal transplantation" OR FMT OR "microbiota transfer" OR "microbiome transfer" OR "fecal transfer" OR "microflora transfer" OR "flora transfer" OR "antibiotic-treated" OR "antibiotic pre-treatment" OR "antibiotic cocktail" OR "antibiotic depletion" OR "microbiota-depleted" OR "microbiome-depleted" OR "microflora-depleted" OR "flora-depleted" OR "germ-free" OR "gnotobiotic" OR "causal relationship" OR "causal role" OR causality OR "cause-effect" OR "cause and effect" OR "microbiota-mediated" OR "microbiome-mediated" OR "microflora-mediated" OR "flora-mediated" OR "microbiota-dependent" OR "microbiome-dependent" OR "microflora-dependent" OR "flora-dependent" OR cohous* OR "co-hous*" OR cohabit* (All Fields) and Article (Document Types) and English (Languages)	179

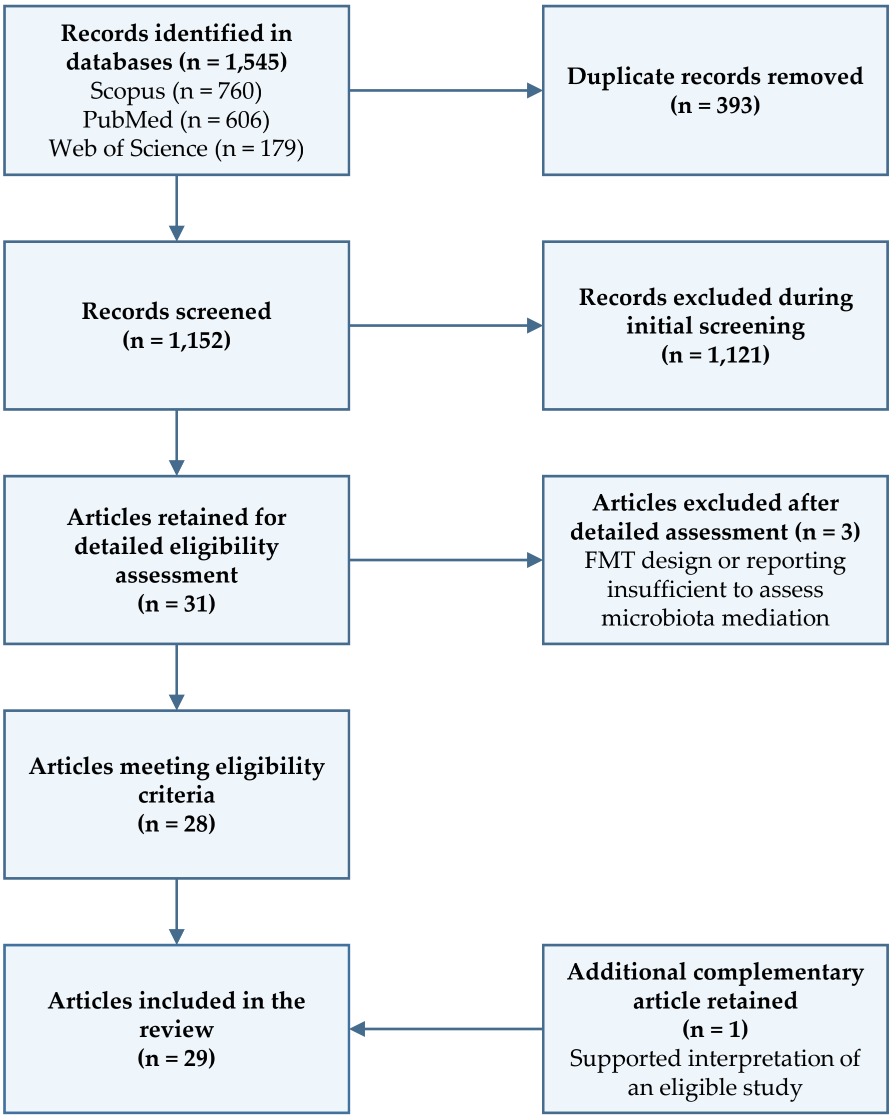

Figure S1. Study selection flow diagram.
